# Whole Transcriptome Analysis Reveals That *Filifactor alocis* Modulates TNFα-Stimulated MAPK Activation in Human Neutrophils

**DOI:** 10.3389/fimmu.2020.00497

**Published:** 2020-04-16

**Authors:** Irina Miralda, Aruna Vashishta, Max N. Rogers, Eric C. Rouchka, Xiaohong Li, Sabine Waigel, Richard J. Lamont, Silvia M. Uriarte

**Affiliations:** ^1^Department of Microbiology and Immunology, School of Medicine, University of Louisville, Louisville, KY, United States; ^2^Department of Medicine, School of Medicine, University of Louisville, Louisville, KY, United States; ^3^Department of Oral Immunology and Infectious Diseases, School of Dentistry, University of Louisville, Louisville, KY, United States; ^4^Department of Biology, School of Arts and Sciences, University of Louisville, Louisville, KY, United States; ^5^Department of Computer Science and Engineering, University of Louisville, Louisville, KY, United States; ^6^KBRIN Bioinformatics Core, University of Louisville, Louisville, KY, United States; ^7^Department of Anatomical Sciences and Neurobiology, University of Louisville, Louisville, KY, United States; ^8^Department of Medicine, University of Louisville Genomics Facility, Louisville, KY, United States

**Keywords:** human neutrophils, periodontitis, emerging oral pathogens, MAPK signaling, neutrophil transcriptome

## Abstract

Periodontitis is an irreversible, bacteria-induced, chronic inflammatory disease that compromises the integrity of tooth-supporting tissues and adversely affects systemic health. As the immune system's first line of defense against bacteria, neutrophils use their microbicidal functions in the oral cavity to protect the host against periodontal disease. However, periodontal pathogens have adapted to resist neutrophil microbicidal mechanisms while still propagating inflammation, which provides essential nutrients for the bacteria to proliferate and cause disease. Advances in sequencing technologies have recognized several newly appreciated bacteria associated with periodontal lesions such as the Gram-positive anaerobic rod, *Filifactor alocis*. With the discovery of these oral bacterial species, there is also a growing need to assess their pathogenic potential and determine their contribution to disease progression. Currently, few studies have addressed the pathogenic mechanisms used by oral bacteria to manipulate the neutrophil functional responses at the level of the transcriptome. Thus, this study aims to characterize the global changes at the gene expression level in human neutrophils during infection with *F. alocis*. Our results indicate that the challenge of human neutrophils with *F. alocis* results in the differential expression of genes involved in multiple neutrophil effector functions such as chemotaxis, cytokine and chemokine signaling pathways, and apoptosis. Moreover, *F. alocis* challenges affected the expression of components from the TNF and MAPK kinase signaling pathways. This resulted in transient, dampened p38 MAPK activation by secondary stimuli TNFα but not by fMLF. Functionally, the *F. alocis*-mediated inhibition of p38 activation by TNFα resulted in decreased cytokine production but had no effect on the priming of the respiratory burst response or the delay of apoptosis by TNFα. Since the modulatory effect was characteristic of viable *F. alocis* only, we propose this as one of *F. alocis'* mechanisms to control neutrophils and their functional responses.

## Introduction

Periodontitis is a chronic inflammatory disease where inflammophilic pathogenic bacterial communities accumulate at the gingival crevice. These dysbiotic microbial communities induce a severe inflammatory response that fails to control bacterial growth and contributes to the irreversible destruction of tooth-supporting tissues (1). Historically, periodontal research has focused on the pathogenic members of the “red complex,” which includes *Porphyromonas gingivalis, Treponema denticola*, and *Tannerella forsythia*. However, recent human microbiome studies have revealed many previously uncultured organisms with a strong correlation with periodontal disease (2, 3). One of these newly appreciated species is *Filifactor alocis*, a Gram-positive anaerobic rod with emerging pathogenic potential and contribution to periodontal diseases. *F. alocis* is consistently and abundantly found in periodontal active lesions (4–9). Furthermore, *F. alocis* shares virulence characteristics with other periodontal pathogens such as resistance to oxidative stress, biofilm formation, secretion of proteases, and evasion of the immune system (10–14).

Neutrophils constitute an overwhelming majority of the leukocytes recruited to the oral cavity, where they are essential for maintaining homeostasis of periodontal tissues (15–17). Neutrophils can deploy several strategies to efficiently detect, detain, and destroy microbes. These include phagocytosis, release of antimicrobial enzymes or toxic factors, generation of massive amounts of reactive oxygen species (ROS), and discharge of their nuclear material into neutrophil extracellular traps (NETs) (18). However, oral pathogens have evolved mechanisms to manipulate neutrophil functional responses to prevent being killed while propagating inflammation (17, 19). Previous work from our laboratory has shown that despite efficient phagocytosis by neutrophils, *F. alocis* survives within neutrophils by inducing minimal production of intracellular ROS and curtailing the fusion of antimicrobial granules with its phagosome (20, 21). However, in comparison to the keystone oral pathogen, *P. gingivalis*, and another emerging oral pathogen, *Peptoanaerobacter stomatis*, challenge with *F. alocis* resulted in a mild release of neutrophil-derived pro-inflammatory cytokines, which resulted in limited recruitment of monocytes and other neutrophils (22). Thus, we hypothesize that *F. alocis* may modulate neutrophil signaling events to interrupt pro-inflammatory cytokine production and alter immune cell recruitment and communication.

The mitogen-activated protein kinases (MAPKs) are evolutionarily conserved regulators that carry out signal transduction for many cellular functional processes. MAPK activation cascades are well-characterized and usually begin with the ligation of cell surface receptors followed by activation of a relay cascade of phosphorylation of three core kinases: MAP3K, MAP2K (MEK or MKK), and MAPK. Active MAPKs can phosphorylate a variety of intracellular targets including transcription factors, nuclear pore proteins, membrane transporters, cytoskeletal elements, and other protein kinases, so their activation is subjected to spatiotemporal regulation by complex feedback and crosstalk mechanisms (23, 24). In human neutrophils, bacterial lipopolysaccharide (LPS) activates Toll-like receptor (TLR) 4 followed by downstream activation of MAPK signaling pathways and the transcription factor regulator nuclear factor (NF)-κB, both of which can independently regulate the production of inflammatory cytokines and chemokines (25, 26). Both p38 MAPK and ERK pathways control transcription and translation of inducible cytokines in neutrophils stimulated with LPS or TNFα (27). Due to the relevant role that MAPK signaling plays in regulation of immune responses, it is not surprising that some pathogens have developed mechanisms to hijack this signaling cascade on immune cells (28, 29). For example, *Mycobacterium tuberculosis* acetylates a MAPK phosphatase, DUSP16, to increase phosphatase activity on Janus kinase (JNK) and limit inflammatory cytokine production by bone marrow-derived macrophages (30). Prior work from our group showed that *F. alocis* initially activates both p38 MAPK and ERK1/2 through TLR2 (20); however, it is unknown what the MAPK response is after *F. alocis* stimulation for longer time points or how the cells respond to secondary stimuli after *F. alocis* challenge.

Few sequencing studies have tracked transcriptome changes in human neutrophils during challenge with a bacterial pathogen (31–34). Even fewer studies have measured changes in the neutrophil transcriptome associated with the challenges of putative oral pathogens. Thus, we sought to characterize global changes at the gene expression level in human neutrophils during infection with *F. alocis*. Analysis of whole-transcriptome by RNA-based next-generation sequencing (RNAseq) shows that *F. alocis* challenge alters the human neutrophil transcriptome by inducing significant changes in the expression of genes involved in various neutrophil effector functions. One of the findings of our RNA-seq screen was that *F. alocis* challenge affected the expression of components in both the TNF and MAPK kinase signaling pathways. This resulted in decreased p38 MAPK activation by secondary stimuli TNFα but not by fMLF. Moreover, only live *F. alocis* limited the TNFα-stimulated production of IL-8, demonstrating that this is one of the mechanisms actively induced by the oral pathogen to control neutrophil functional responses.

## Materials and Methods

### Human Neutrophil Isolation

Human donor recruitment, blood draws, and the use of the materials required for this procedure were done in accordance with the guidelines approved by the Institutional Review Board of the University of Louisville. Neutrophils were isolated from venous blood of healthy donors using plasma-Percoll gradients as described previously (35). Neutrophil populations were further enriched to obtain highly pure cells (>99%) by negative magnetic selection using the Easy Eights EasySep Magnet and human neutrophil enrichment kit (Stemcell Technologies, Vancouver, BC, Canada) as previously described (36). Cell purity was assessed by simultaneously staining with FITC-conjugated anti-CD66b (clone G10F5; BioLegend, San Diego, CA, USA) and APC-conjugated anti-CD16 (clone CB16; eBioscience, San Diego, CA, USA) antibodies and determining the percentage of CD66b^+^CD16^+^ cells using a BD Celesta flow cytometer (BD Biosciences, San Jose, CA, USA). Both pure (>90–95%) and highly pure (>99%) neutrophils were cultured in complete RPMI-1640 medium (Sigma-Aldrich, St. Louis, MO, USA) with 5% human serum (Atlanta Biologicals, Flowery Branch, GA, USA).

### Bacterial Strains and Growth Conditions

*F. alocis* ATCC 38596 was cultured in brain heart infusion (BHI) broth supplemented 5 mg/mL yeast extract, L-cysteine (0.05%), and arginine (0.05%) for 7 days anaerobically at 37°C as previously described (20, 37). Serum opsonization was performed by incubating *F. alocis* at 37°C for 20 min in 10% normal human serum (Complement Technology, Inc., Tyler, TX, USA). Heat-killed *F. alocis* was generated by incubation at 90°C for 60 min. Non-viability was confirmed by incubation in culture media at the same conditions used for the live organism.

### *F. alocis* Challenge and RNA Isolation

Highly pure (>99%) human neutrophils (10–20 × 10^6^ cells/mL) were unstimulated or challenged with opsonized *F. alocis* at a multiplicity of infection (MOI) of 10, for 1, 3, or 6 h. The infection was synchronized by centrifugation at 14°C for 4 min at 600 × g. After each time point, the cells were harvested using Trizol (Life Technologies, Carlsbad, CA, USA) and stored at −80°C until RNA extraction. RNA was isolated from unstimulated and *F. alocis*-challenged human purified neutrophils using the hybrid method of Trizol and RNeasy minikit (Qiagen, Venlo, Netherlands). The aqueous phase containing RNA was loaded on the Qiagen column for further purification of RNA. The purified RNA quality was measured by running the sample on Bioanalyzer.

### Library Preparation

The isolated RNA was checked for integrity using the Agilent Bioanalyzer 2100 system (Agilent Technologies, Santa Clara, CA, USA) and quantified using a Qubit fluorometric assay (Thermo Fisher Scientific, Waltham, MA, USA). Total RNASeq libraries were prepared following Illumina's TruSeq Stranded Total RNA LT with Ribo-Zero Gold library preparation protocol (Illumina Inc., San Diego, CA, USA, Cat# RS-122-2301). After depletion of ribosomal RNA, all samples were ligated with Illumina adapters and individually barcoded. The absence of adapter dimers and a consistent library size of approximately 300 bp was confirmed using the Agilent Bioanalyzer 2100. Library quantitation was performed by qPCR using the KAPA Library Quantitation Kit (Kapa Biosystems, part of Roche Sequencing and Life Science, Wilmington, MA, USA) for Illumina Platforms.

### Sequencing Run

1.8 pM of the library pool was loaded with 1% PhiX spike-in on two NextSeq 500/550 75 cycle High Output Kit v2 sequencing flow cells. Sequencing was performed on the Illumina NextSeq 500 sequencer targeting 50M 1 × 75bp reads per sample.

### Bioinformatic Analysis

Each of four single-end raw FASTQ files for each replicate was concatenated into one single end FASTQ file using the Unix cat command. A total of sixteen files representing four independent donors and four experimental conditions were generated. Quality control (QC) of the raw sequence data was performed using FastQC (version 0.10.1). The interquartile range remained above 30 (99.9% base call accuracy) across the reads. The concatenated sequences were directly aligned to the *Homo sapiens* reference genome assembly (hg38.fa) using tophat2 (version 2.0.13) (38), generating alignment files in BAM format. The alignment rate ranged from 88 to 93 percent across the samples. Differential expression analysis between each treatment condition (1, 3, and 6 h) and the control condition was performed using Cufflinks-Cuffdiff2 (version 2.2.1) (39, 40). A q-value cutoff ≤ 0.05 with an absolute |log_2_FC| ≥ 1 was used to determine differential expression.

### Reverse Transcription and Quantitative Real-Time PCR (RT-qPCR)

Total RNA isolated from the different experimental conditions was followed by reverse-transcription into cDNA using a High Capacity RNA to cDNA kit (Applied Biosystems, Foster City, CA, USA), while qPCR was carried out using SYBR®Green PCR Master Mix (Applied Biosystems, Foster City, CA, USA) on an Applied Biosystems StepOnePlus cycler with StepOne software V2.2.2. Sequences of the gene-specific primers (Integrated DNA Technologies, Skokie, IL, USA) used in this study are listed in Table 1. Data were calculated and expressed as mean normalized expression (MNE) units after GAPDH normalization as previously described (41).

**Table 1:** qPCR primer sequences used to validate RNAseq results.

**Gene**	**qPCR primer sequence**
Galectin 3	Forward 5′- CAGAATTGCTTTAGATTTCCAA-3′
	Reverse 5′-TTATCCAGCTTTGTATTGCAA-3′
NCF-1	Forward 5′-AAGATGGCAAGAGTACCGC-3′
	Reverse 5′-TCTCGTAGTTGGCAATGGC-3′
GAPDH	Forward 5′-CTTTGGTATCGTGGAAGGACTC-3′
	Reverse 5′-GTAGAGGCAGGGATGATGTTC-3′
CXCL5	Forward 5′-TCTGCAAGTGTTCGCCATAG-3′
	Reverse 5′-CAGTTTTCCTTGTTTCCACCG-3′
CCL5	Forward 5′-TGCCCACATCAAGGAGTATTT-3′
	Reverse 5′-TTTCGGGTGACAAAGACGA-3′
ASC	Forward 5′-CTCCTCAGTCGGCAGCCAAG-3′
	Reverse 5′-ACAGAGCATCCAGCAGCCAC-3′
NOD2	Forward 5′-CTGAAGAATGCCCGCAAGGT-3′
	Reverse 5′-GTCTCTTGGAGCAGGCGGATG-3′

### Western Blotting

Neutrophils (10 × 10^6^ cells/mL) were cultured at 37°C, 5%CO_2_ in RPMI-1640 with 5% heat-inactivated human serum and left unstimulated, stimulated with FSL (100 ng/mL), challenged with either live or heat-killed *F. alocis* for 1, 3, 6, or 10 h followed by stimulation with fMLF (300 nM, 1 min) or TNF-α (10 ng/ml, 15 min). After the different experimental procedures, cells were centrifuged at 6,000 × g for 30 s and lysed for 30 min on ice in ice-cold lysis buffer [20 mM Tris-HCl [pH 7.5], 150 mM NaCl, 1% [vol/vol] Triton X-100, 0.5% [vol/vol] Nonidet P-40, 20 mM NaF, 20 mM NaVO3, 1 mM EDTA, 1 mM EGTA, 5 mM phenylmethylsulfonyl fluoride [PMSF], 21 μg/ml aprotinin, 5 μg/ml leupeptin, and 4 mM Diisopropyl fluorophosphates [DFP]]. After protein estimation using the Pierce BCA Protein Assay Kit (Thermo Scientific, Waltham, MA, USA), samples were adjusted to a concentration of 2 μg/μL. 16–20 μg/μL of total cell lysates were separated by 12% SDS-PAGE and immunoblotted with antibodies for phospho-ERK1/2, total ERK1/2, phospho-p38 MAPK, total p38 MAPK, phospho-AKT, total AKT, phospho-S6 (Cell Signaling Danvers, MA, USA), p47phox, or p67phox (gift from Dr. William M. Nauseef), all at 1:1,000 dilution. The appropriate secondary antibodies were used at 1:2,000 dilution (Cell Signaling, Danvers, MA, USA). The ECL System (Amersham Pharmacia Biotech, Little Chalfont, United Kingdom) or the SuperSignal West Femto Maximum Sensitivity Substrate (Thermo Scientific, Waltham, MA, USA) was used to visualize antigen-antibody reactions. Densitometric values of each band were calculated using Image Lab Software (BioRad, Hercules, CA, USA).

### Superoxide Generation and Priming

Superoxide anion release was measured spectrophotometrically at 550 nm as the superoxide dismutase-inhibitable reduction of ferricytochrome c as previously described (35). Briefly, neutrophils (4 × 10^6^cells/ml) were cultured in RPMI supplemented with 5% heat-inactivated human serum and left untreated or pre-treated with p38 inhibitor BIRB-796 (75 nM, added to media 60 min before 6- and 10-h time points; Sigma, St. Louis, MO, USA), or with opsonized *F. alocis* (MOI 10) for 6 and 10 h at 37°C in a shaking water bath. After this first pre-treatment, TNFα (10 ng/ml, 10 min) was added to all the samples. Samples were run in duplicate, with one duplicate used to detect basal superoxide levels in the presence or absence of each pre-treatment and the other duplicate used to measure TNF-priming by further challenge with fMLF (300 nM) for 5 min. After stimulation of superoxide production, the samples were centrifuged for 10 min at 600 × g, 4°C, supernatants were collected, and optical densities were read.

### IL-8 Cytokine Measurement and Apoptosis

Neutrophils (10 × 10^6^ cells/mL) were cultured in RPMI + 5% heat-inactivated human serum and left untreated, or pre-treated with TAK1 inhibitor (5Z)-7-Oxozeanol (3 μM, added 30 min before 6- and 10-h time points; Cayman, Ann Arbor, MI, USA) p38 inhibitor BIRB-796 (75 nM, added 60 min before 6- and 10-h time points; Sigma, St. Louis, MO, USA), or opsonized *F. alocis* (MOI 10) for 6 and 10 h at 37°C in an incubator with 5% CO_2_. After the pre-treatment, the volume in the tube was divided evenly between two tubes, with tube receiving TNFα (10 ng/ml) and the other nothing. All tubes were returned to the incubator for 4 or 12 h. After the TNFα stimulation period, cells were centrifuged, their supernatants collected, and the pellets tested for apoptosis. 1% protease and phosphatase inhibitors were added to the supernatants to protect them from degradation. IL-8 was measured in the supernatants using a commercially available kit (Invitrogen, Carlsbad, CA, USA). Cells were processed for Annexin V/7-AAD staining using the commercially available APC Annexin V Apoptosis Detection Kit with 7-AAD (BioLegend, San Diego, CA, USA). Samples were read on a BD FACSCelesta flow cytometer and analyzed using the FlowJo software (Ashland, OR, USA).

### Statistical Analysis

Unless otherwise noted, statistical differences among experimental conditions and time points were analyzed by a repeating measures two-way ANOVA, followed by Bonferroni post-tests using GraphPad Prism Software (Graphpad San Diego, CA, USA). Differences were considered significant at the level *P* < 0.05. When a two-way ANOVA was not applicable, a one-way ANOVA followed by the *post hoc* Tukey's multiple-comparison test was used.

## Results

### *F. alocis* Induces Global Changes in Gene Expression

**Figure 1:**
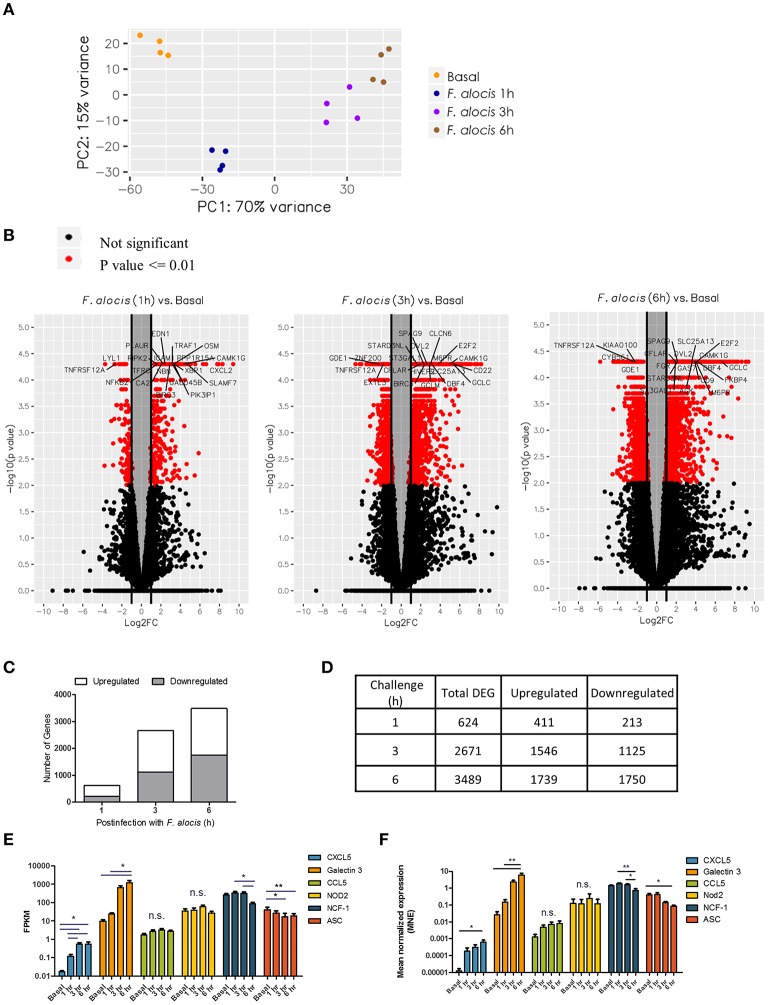
Global changes in the transcriptome of *F. alocis*-challenged neutrophils. Illumina RNA sequencing was performed on human neutrophils from four individual healthy donors that were either left unstimulated (basal) or challenged with *F. alocis* for 1, 3, or 6 h. PCA analysis shows the variation between the four donors at each time point **(A)**. The Tuxedo Suite program Cuffdiff2 was used to acquire a list of differentially expressed genes (DEG) that were graphed in volcano plots **(B)**. Genes that had *p* ≤ 0.01 and log_2_ FC| ≥ 1 are colored in red, and genes with high fold changes are labeled. For each time point, the average number of upregulated and downregulated differentially expressed genes (DEG) was determined and plotted **(C,D)**. Six genes (two upregulated, two with no change, and two downregulated) were chosen to validate the RNAseq data by quantitative qPCR analysis. From the RNAseq data, the fragments per kilobase of transcript per million mapped reads (FPKM) for these six genes are plotted as mean ± SEM from the four donors **(E)**. From the qPCR analysis, the mean normalized mRNA expression (MNE) from five independent experiments are plotted in **(F)** as MNE ± SEM. One-way ANOVAs were performed on the expression levels from each gene to determine statistical significance between the basal condition and each time point. n.s.: non-significant, *p < 0.05, **p < 0.01.

To assess changes in gene expression after *F. alocis* challenge, whole transcriptome by RNA-based next-generation sequencing (RNA-seq) was performed on human neutrophils from four individual healthy donors that were either left unstimulated or challenged with *F. alocis* for 1, 3, or 6 h. All time points for each donor were mapped onto a principal component analysis (PCA) plot to determine the variation in the dataset (Figure 1A). All four donors clustered together for each experimental condition, showing that donor variability is not a major contributor in our dataset. Contrastingly, the transcriptional profile of *F. alocis*-stimulated neutrophils clearly separated from basal conditions at each time point. Next, differential expression analysis was completed between each treatment condition using the Tuxedo Suite Program Cuffdiff2, where a *p*-value cutoff ≤ 0.01 and a log fold change |log_2_FC| ≥ 1 was utilized to compile a list of differentially expressed genes (DEG) for further analyses. Volcano plots for each time point showed that *F. alocis* challenge induced a time-dependent change in gene expression, as the number of red-colored dots, which represent the significant DEG, grew at each time point (Figure 1B). On the volcano plots, the most significant DEG for each time point was identified. Out of these highlighted DEGs, *CAMK1G* was upregulated and *TNFRSF12A* was downregulated at all three time points compared to unstimulated cells. The *CAMK1G* gene encodes a protein like calcium/calmodulin-dependent protein kinase; however, according to RefSeq, its exact function is unknown. *TNFRSF12A*, also known as Fn14, is a weak inducer of apoptosis that can activate NF-kB signaling pathways, promote oxidative stress, and is linked to high expression of matrix metalloprotease 9 (MMP-9) (42–45).

The number of DEGs was determined for each time point and plotted based on whether they were upregulated or downregulated compared to the basal control (Figure 1C). The biggest change in transcriptome occurred early in the time course, with 624 genes differentially expressed at 1 h and a steep increase in the number of DEGs between 1 and 3 h (Figure 1D). At the 6-h time point, the number increased only marginally from 3 h. Throughout the time course, the number of genes induced was roughly the same as the number of repressed genes. While our DEG criteria are more stringent than other studies, these global changes in gene expression appear to be unique to *F. alocis* as compared to other transcriptome studies between neutrophils and bacterial challenge (31–34). To validate the RNAseq data, two upregulated genes, two downregulated genes, and two genes with no change were selected for validation by quantitative PCR. Figure 1E shows the fragments per kilobase of transcript per million mapped reads (FPKM) expression values for all four donors from the RNAseq screen, while Figure 1F shows the mean normalized mRNA expression by qPCR. Overall, the qPCR results validate the RNAseq screen and provide confidence about the targets identified by the high throughput screening analysis.

### *F. alocis* Affects Neutrophil Functional and Biochemical Processes

To reduce bias during the bioinformatic analysis, the DEG list was uploaded into two separate databases: Database for Annotation, Visualization and Integrated Discovery (DAVID) (46, 47) and MetaCore by Clarivate Analytics. In each database, we first identified the significant (p < 0.01) biological processes during challenge with *F. alocis*. From the DAVID analysis, 37, 74, and 86 processes were identified for the 1-, 3-, and 6-h time points, respectively, and categorized by cell function (Supplemental Table 1). Significant process categories in every time point include biological processes related to the inflammatory response, response to microbes, chemotaxis, signal transduction, gene expression and transcription factor regulation, cytokine-mediated responses and production, and apoptosis. However, as the time course progressed, there was a shift in the affected processes. While cytokine-related processes were most prominent at the earlier time points, biological processes related to phagosome maturation and metabolic processes became significant at the later time points. Moreover, processes related to protein folding only became significant at 6 h post-bacterial challenge.

**Figure 2:**
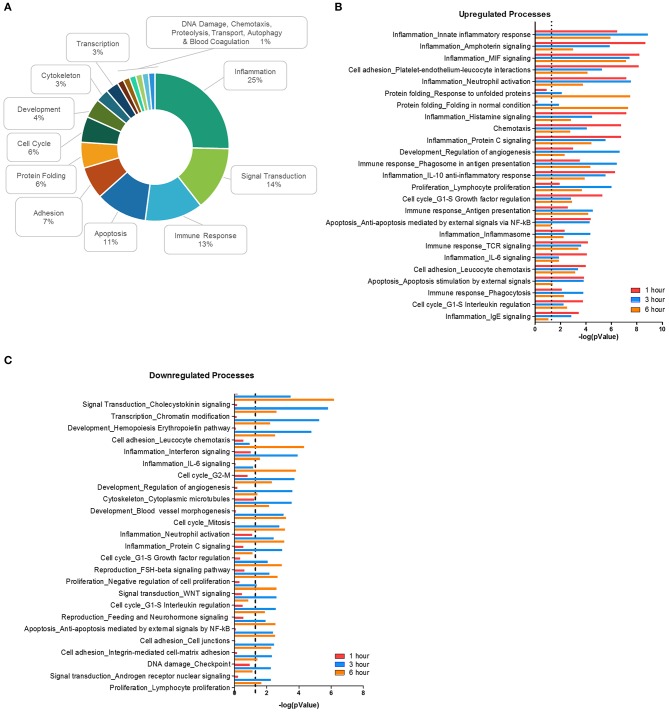
Biological processes affected during *F. alocis* challenge. The list of differentially expressed genes (DEG) for each time point was input to the proprietary, manually curated MetaCore database and analyzed for biological processes that contained a significant number of DEG. MetaCore process networks are categorized by cell functions, so the frequency of each cell function was tallied for all the 71 significant (*p* < 0.01) processes. Percentages for each function are displayed as a donut graph **(A)**. The top 25 upregulated **(B)** or downregulated **(C)** network processes were then graphed according to the –log of the *p* value for each time point. The dotted line represents a *p* value of 0.01.

Using MetaCore, 71 significant (*p* < 0.01) network processes were determined in our data set. Since the MetaCore software automatically categorizes the processes by cell function, we determined the frequency of each category (Figure 2A). Like the DAVID analysis, most processes were involved with inflammation, signal transduction, the immune response, and apoptosis. Cell function processes with a lower frequency include protein folding, cytoskeleton, transcription, chemotaxis, and autophagy. Next, we plotted the top 25 most significantly upregulated (Figure 2B) and downregulated processes (Figure 2C). Processes related to inflammation made up four out of the top five upregulated network processes, but the significance of the inflammatory processes decreased as the time course progressed. In fact, some of these inflammation processes from the upregulated list became significant in the downregulated processes during the later time points, as is the case with processes such as IL-6 signaling and neutrophil activation (Supplemental Table 2). This suggests that *F. alocis* may be dampening inflammatory processes between 1 and 3 h to prolong its survival or provide protection to bystander species. Together, this data shows that *F. alocis* challenge induces temporal changes in neutrophil functional mechanisms like cytokine production, chemotaxis, vesicular trafficking, and degranulation, as well as neutrophil biochemical mechanisms like the regulation of signaling pathways and metabolism. This coincides with previous data from our laboratory that shows that *F. alocis* affects neutrophil cytokine production, chemotaxis, vesicle trafficking, and degranulation functions (21, 22).

Based on previous work that demonstrated that *F. alocis* induces minimal intracellular and extracellular ROS production (21), we looked at whether the components of the NAPDH oxidase complex are affected during *F. alocis* challenge (Table 2). From the RNAseq data, the only statistically significant results show that the expression of CYBB (gp91phox subunit) increased in a time-dependent manner while the expression of NCF1 (p47phox subunit) decreased by 6 h of challenge, which was also validated by qPCR (Figure 1F). While the minimal ROS activation at the early time points cannot be attributed to modulation of gene expression by *F. alocis*, generation of ROS at later time points may be inhibited by the expression of a member of the galectin family of carbohydrate-binding proteins, galectin-3. The increased expression of galectin-3 has already been associated with inhibition of ROS production when human neutrophils were challenged with *Candida albicans* (48). In our dataset, both the FPKM expression as well as the qPCR validation show a time-dependent increase in galectin-3 mRNA expression (Figures 1E,F), and when tested by western blot, *F. alocis* induced a time dependent increase in galectin-3 protein expression (data not shown).

**Table 2:** Fold change of Components of NADPH Oxidase Complex compared to basal.

**Ensembl Gene**	**Gene Symbol|Description**	**1 h**	**3 h**	**6 h**
ENSG00000165168	CYBB|cytochrome b-245, beta polypeptide	1.4	3.04*	3.09*
ENSG00000051523	CYBA|cytochrome b-245, alpha polypeptide	0.9	1.4	1.1
ENSG00000158517	NCF1|neutrophil cytosolic factor 1	1.3	1.2	0.32*
ENSG00000116701	NCF2|neutrophil cytosolic factor 2	1.0	1.5	1.4
ENSG00000100365	NCF4|neutrophil cytosolic factor 4, 40kDa	1.0	0.8	1.1
ENSG00000128340	RAC2|ras-related C3 botulinum toxin substrate 2 (rho family, small GTP binding protein Rac2)	0.9	1.4	1.3
ENSG00000116473	RAP1A|RAP1A, member of RAS oncogene family	1.1	0.8	0.9

*Asterisk denotes p < 0.05 compared to basal unstimulated conditions*.

### *F. alocis* Challenge Upregulates Cytokine Pathways and Downregulates Signaling Pathways

Next, we identified pathways relevant to challenge with *F. alocis*. Using DAVID, the DEG list was mapped onto predefined pathways from the Kyoto Encyclopedia of Genes and Genomes (KEGG) database. We limited our analysis to highly significant pathways with a *p* < 0.01, which resulted in 10, 26, and 33 pathways for the 1-, 3-, and 6-h time points, respectively (Table 3). The *F. alocis*-neutrophil transcriptome reinforced the pathogenic potential of *F. alocis* by the number of significant pathways linked to pathogens that subvert immune cells (*Salmonella, Legionella, Helicobacter pylori*, and Influenza A). Similarly, pathways for cancers, rheumatoid arthritis, and inflammatory bowel disease were significant for the *F. alocis-*challenged neutrophil transcriptome in both databases. Oral bacteria continue to be linked to systemic malignancies like those listed above (49), and although *F. alocis* has not been amongst the oral pathogens detected yet, these results hint that it could play a role in the pathogenesis of these diseases.

**Table 3:** KEGG pathways significantly enriched for differentially expressed genes during challenge with *F. alocis*.

**Time point**	**DEG count**	**Description**	***P*-value**
1 h	19	Cytokine-cytokine receptor interaction	5.40E-09
	13	TNF signaling pathway	2.50E-08
	10	Rheumatoid arthritis	3.50E-06
	9	NF-kappa B signaling pathway	2.70E-05
	8	Salmonella infection	1.50E-04
	6	Legionellosis	9.20E-04
	6	NOD-like receptor signaling pathway	1.10E-03
	9	Transcriptional mis-regulation in cancer	2.40E-03
	9	Chemokine signaling pathway	4.60E-03
	10	MAPK signaling pathway	9.10E-03
3 h	26	Rheumatoid arthritis	2.30E-08
	19	Legionellosis	1.50E-07
	29	Lysosome	4.10E-07
	26	TNF signaling pathway	1.40E-06
	18	NOD-like receptor signaling pathway	1.50E-06
	22	NF-kappa B signaling pathway	5.70E-06
	40	Cytokine-cytokine receptor interaction	4.00E-05
	17	Epithelial cell signaling in Helicobacter pylori infection	8.50E-05
	19	Salmonella infection	1.20E-04
	22	Toll-like receptor signaling pathway	1.30E-04
	30	Chemokine signaling pathway	6.40E-04
	20	Chagas disease (American trypanosomiasis)	8.00E-04
	13	Vibrio cholerae infection	9.00E-04
	28	Influenza A	1.00E-03
	23	Measles	1.30E-03
	34	Endocytosis	2.70E-03
	24	Phagosome	2.80E-03
	13	Apoptosis	4.40E-03
	20	Epstein-Barr virus infection	5.30E-03
	13	Inflammatory bowel disease (IBD)	5.70E-03
	17	Inflammatory mediator regulation of TRP channels	6.40E-03
	26	Herpes simplex infection	8.70E-03
	10	Sphingolipid metabolism	0.014
	12	Pancreatic cancer	0.017
	32	HTLV-I infection	0.018
	27	Viral carcinogenesis	0.019
6 h	22	Legionellosis	2.70E-07
	46	Chemokine signaling pathway	1.20E-06
	20	Apoptosis	1.80E-05
	30	Lysosome	1.20E-04
	32	Measles	1.20E-04
	37	Influenza A	4.40E-04
	24	Estrogen signaling pathway	9.10E-04
	22	Rheumatoid arthritis	1.00E-03
	40	Viral carcinogenesis	1.40E-03
	16	NOD-like receptor signaling pathway	1.60E-03
	45	Endocytosis	1.80E-03
	29	Insulin signaling pathway	2.50E-03
	24	TNF signaling pathway	2.70E-03
	26	Sphingolipid signaling pathway	2.90E-03
	8	Other glycan degradation	3.00E-03
	26	Epstein-Barr virus infection	3.60E-03
	17	Epithelial cell signaling in Helicobacter pylori infection	4.00E-03
	24	Toxoplasmosis	4.00E-03
	23	Chagas disease (American trypanosomiasis)	4.10E-03
	23	Toll-like receptor signaling pathway	5.20E-03
	13	Sphingolipid metabolism	6.80E-03
	16	Pancreatic cancer	7.30E-03
	33	Tuberculosis	8.20E-03
	21	Phosphatidylinositol signaling system	9.10E-03
	21	Inflammatory mediator regulation of TRP channels	9.10E-03
	14	Non-small cell lung cancer	0.011
	17	Pertussis	0.012
	19	Prostate cancer	0.013
	13	Vibrio cholerae infection	0.015
	25	Platelet activation	0.016
	16	Leishmaniasis	0.016
	27	Hepatitis B	0.018
	11	Bladder cancer	0.018

**Figure 3:**
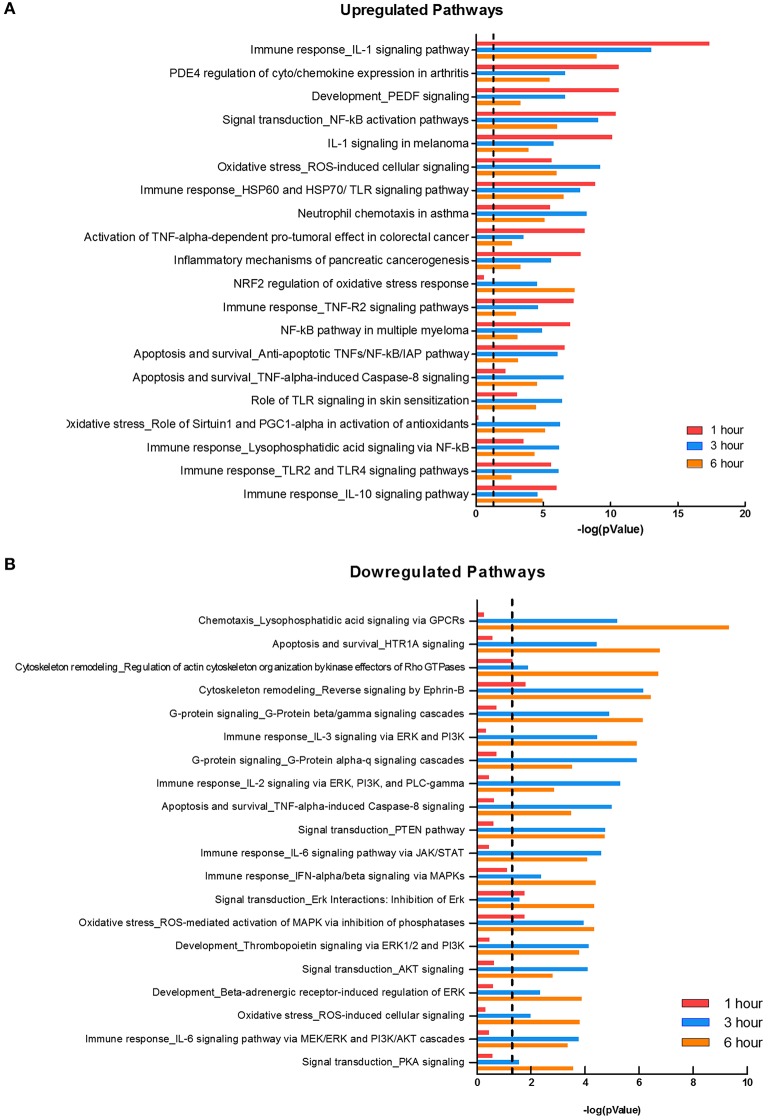
Pathways affected during *F. alocis* challenge. Using the MetaCore database, we determined the top 20 upregulated **(A)** or downregulated **(B)** pathways in the transcriptome of *F. alocis*-challenged neutrophils. These pathways are graphed according to the –log_10_ of the *p-*value for each time point. The dotted line represents a *p-*value of 0.01.

Two major bacterial recognition receptor signaling pathways, NOD-like receptor and Toll-like receptor signaling, were identified in our data set. These receptor pathways align with published data on *F. alocis-*induced cytokine production, where NOD1 is activated during challenge with heat-killed *F. alocis* to produce IL-6 in monocytes (50), and TLR2/6 activation of neutrophils leads to the production and release of cytokines and chemokines (22). Using Metacore, we divided the significant pathways into the top 20 upregulated (Figure 3A) and downregulated (Figure 3B) plots. The list of upregulated pathways supports published data that shows initial contact with *F. alocis* results in the early transcription and production of cytokines (22). At 1 h, cytokine-related pathways such as cytokine-cytokine receptor interaction, TNF signaling pathway, and chemokine signaling pathway were also the most prominent pathways by KEGG analysis (Table 3). In both databases, the NFκB signaling pathway was significant and upregulated, suggesting this transcription factor is likely responsible for the cytokine and chemokine transcriptome response.

While many of the upregulated pathways were related to inflammation and cytokine responses, the downregulated list was largely comprised of signal transduction pathways (Figure 3B). The pathways were significantly affected at the later time points and include signal transduction by MAPK, GPCR, Rho GTPases, PI3K, PTEN, AKT, and PKA. Out of the list of 20 downregulated pathways, seven relate to MAPK signaling. To further support this analysis, under the signal transduction biological process category, positive regulation of ERK1 and ERK2 cascade and activation of MAPK activity are significant at 1 h, but inactivation of MAPK activity becomes significant at 3 and 6 h (Table 3). We focused on this pathway and determined if *F. alocis* is modulating MAPK signaling in human neutrophils.

### *F. alocis* Challenge Does Not Affect fMLF-Stimulated MAPK Signaling

**Figure 4:**
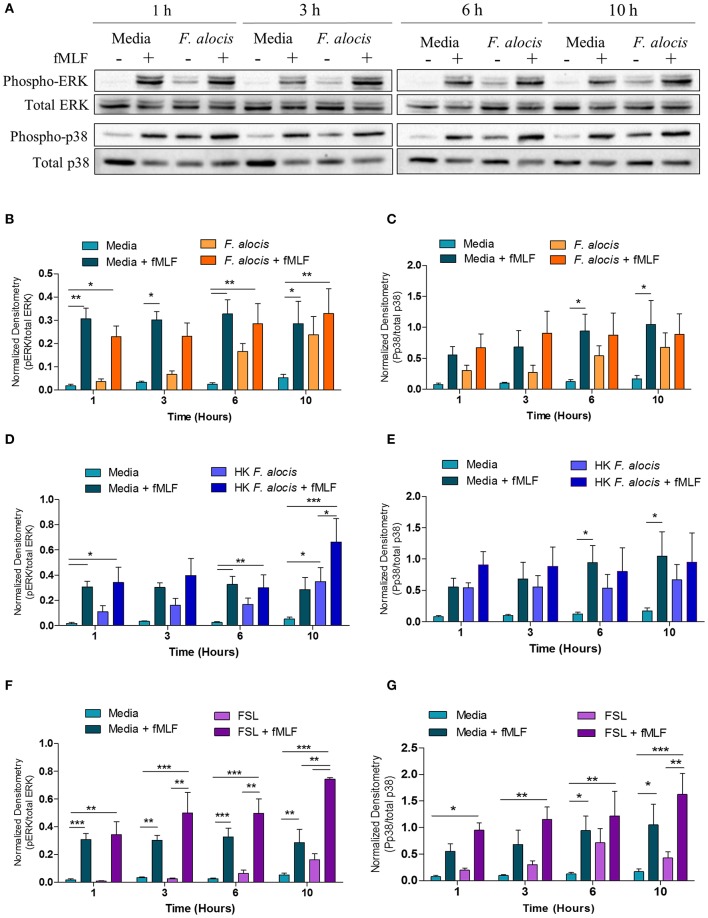
*F. alocis* effect on fMLF-induced MAPK activation. To assess whether *F. alocis* is interfering with MAPK signaling, human neutrophils were cultured in media with or without *F. alocis* at an MOI of 10 for 1, 3, 6, or 10 h followed by stimulation with fMLF for 1 min. Western blots of whole cell lysates were probed for phosphorylated and total p38 and ERK1/2 **(A)** and quantified by densitometry **(B,C)**. Alternatively, neutrophils were cultured in media alone, media with heat-killed *F. alocis* **(D,E)** or the TLR2/6 agonist FSL **(F,G)** for 1, 3, 6, or 10 h before fMLF stimulation. Densitometries are plotted as the mean ± SEM from 6 **(B,C)** and 4 **(D–G)** independent experiments. Statistical differences among experimental conditions and time points were analyzed by a repeating measures two-way ANOVA, followed by Bonferroni post-tests. *p < 0.05, **p < 0.01, ***p < 0.001.

Since G-protein coupled receptor (GPCR) and MAPK were both hits in our dataset, western blots to evaluate ERK1/2 and p38 MAPK activation were performed on lysates from human neutrophils pretreated with media or media containing *F. alocis* for 1, 3, 6, and 10 h followed by stimulation with the bacterial peptide N-Formylmethionine-leucyl-phenylalanine (fMLF) (Figure 4A). Densitometry analysis of the western blots bands for phosphorylated and total ERK1/2 showed that stimulation with *F. alocis* alone has a time-dependent increase in phosphorylation of ERK1/2 (Figure 4B). This suggests there is a bimodal response in the activation of ERK1/2 since it was previously published that ERK phosphorylation peaks at 15 min and then decreases (20). In the case of p38 MAPK, the levels of phosphorylated p38 MAPK are also increased in the *F. alocis* pre-treated cells as compared to neutrophils cultured in media alone (Figure 4C). However, the levels remain steadily elevated throughout the time course. This pattern of phosphorylation for the MAPK is also observed, although at different magnitudes, when neutrophils are pre-treated with heat-killed *F. alocis* (Figures 4D,E) and the TLR2/6 agonist FSL1 (Figures 4F,G). Despite the increased basal levels of phosphorylated ERK1/2 and p38 MAPK in the viable and heat-killed *F. alocis* pretreated cells, when the neutrophils are stimulated with fMLF after pre-treatment with *F. alocis*, the phosphorylation of both ERK and p38 MAPK is comparable to that of cells cultured in media alone (Figures 4B,C). Contrastingly, cells pretreated with FSL1 showed increased phosphorylation of ERK1/2 when stimulated with fMLF, which became significant at 10 h compared to media-cultured cells stimulated with fMLF alone (Figure 4F). A similar trend was observed at the 10-h time point with phosphorylation of p38 MAPK, but the data did not reach statistical significance when compared to fMLF alone (Figure 4G).

### *F. alocis* Challenge Dampens TNF-α-Stimulated MAPK Signaling

**Figure 5:**
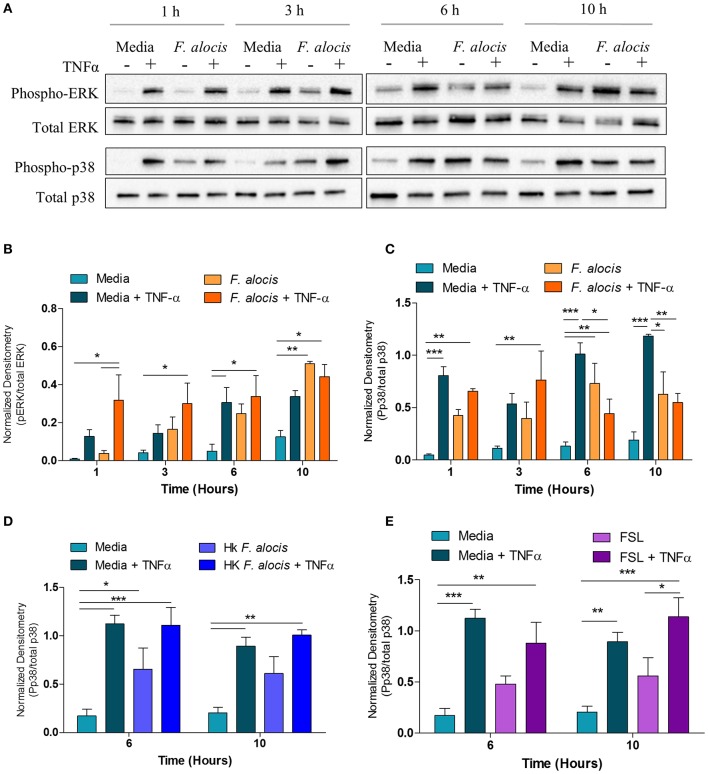
*F. alocis* effect on TNFα-induced MAPK activation. Human neutrophils were cultured in media with or without *F. alocis* at an MOI of 10 for 1, 3, 6, or 10 h followed by stimulation with TNF-α for 15 min. Western blots with whole cell lysates were probed for phosphorylated and total p38 and ERK1/2 **(A)** and quantified by densitometry **(B,C)**. To determine if the decreased p38 phosphorylation at 6 and 10 h was specific to the viable bacteria or a consequence of TLR 2/6 ligation, neutrophils were cultured in media alone, media with heat-killed *F. alocis* **(D)** or the TLR2/6 agonist FSL **(E)** for 6 or 10 h before TNF-α stimulation. Western blots of whole cell lysates were probed for phosphorylated and total p38. Densitometries are plotted as the mean ± SEM from three independent experiments. Statistical differences among experimental conditions and time points were analyzed by a repeating measures two-way ANOVA, followed by Bonferroni post-tests. *p < 0.05, **p < 0.01, ***p < 0.001.

From the DAVID analysis, one of the KEGG pathways that was significantly modulated by *F. alocis* at each time point was the TNF signaling pathway (Table 3). Similarly, four of the upregulated pathways and one of the downregulated pathways from the MetaCore analysis involve TNF signaling (Figures 3A,B). Since it is well-documented that stimuli like LPS and TNFα can activate the p38 and MEK/ERK pathways in neutrophils (25, 51, 52) and high levels of TNFα are present in periodontitis active sites (53, 54), we tested the effect of *F. alocis* pre-treatment on TNFα-induced MAPK signaling cascade. Whole cell lysates from neutrophils pre-treated with media or *F. alocis* followed by stimulation with TNFα were immunoblotted for phosphorylated and total ERK1/2 and p38 MAPK (Figure 5A). Densitometry analysis of the ERK immunoblots showed that pre-treatment with *F. alocis* did not impact TNFα-driven phosphorylation of ERK1/2 (Figure 5B). However, the TNFα-driven phosphorylation of p38 MAPK was significantly dampened in neutrophils pre-treated with *F. alocis* for 6 and 10 h as compared to neutrophils cultured in media alone (Figure 5C). This effect is dependent on the bacteria being viable, because when the neutrophils were pre-treated with heat-killed *F. alocis* before stimulation with TNFα, there was no decrease in the p38 phosphorylation at 6 or 10 h (Figure 5D). Additionally, ligation of TLR2/6 is insufficient to elicit the phenotype observed (Figure 5E). This data shows that viable *F. alocis* modulates TNFα-induced activation of the MAPK signaling pathway by selectively interfering with the phosphorylation of p38 MAPK, but not ERK1/2.

**Figure 6:**
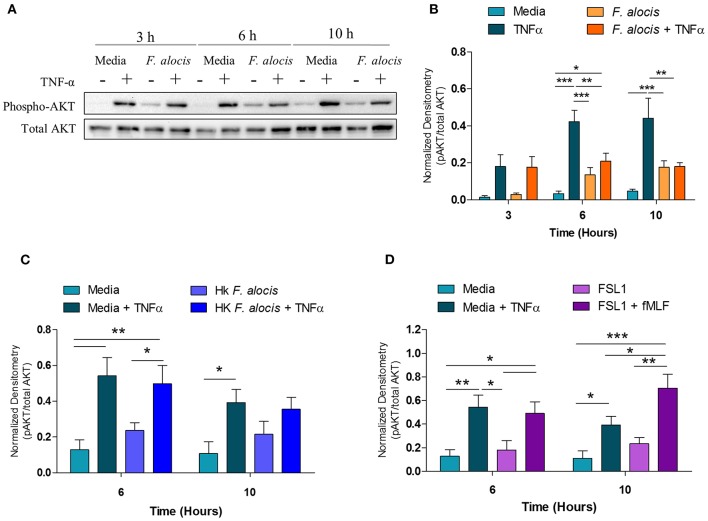
*F. alocis* effect on TNFα-induced AKT activation. Whole cell lysates from human neutrophils pre-treated with media alone or *F. alocis* (MOI 10) for 3, 6, or 10 h followed by TNF-α stimulation were probed for phosphorylated and total AKT **(A)** and quantified by densitometry **(B)**. Similarly, neutrophils were cultured in media alone, media with heat-killed *F. alocis* **(C)** or the TLR2/6 agonist FSL **(D)** for 6 or 10 h before TNF-α stimulation. Densitometries are plotted as the mean ± SEM from 3 **(B)** and 4 **(C,D)** independent experiments. Statistical differences among experimental conditions and time points were analyzed by a repeating measures two-way ANOVA, followed by Bonferroni post-tests. *p < 0.05, **p < 0.01, ***p < 0.001.

In human neutrophils, activation of p38 MAPK by TNFα results in the downstream phosphorylation and activation of AKT (55). Therefore, since the TNFα-induced activation of p38 MAPK was affected when neutrophils were pre-treated with *F. alocis*, the activation of AKT should also be compromised. To test this hypothesis, the lysates from media and *F. alocis* pre-treated neutrophils stimulated with TNFα were immunoblotted for phosphorylated and total AKT (Figure 6A). Densitometry analysis of the Western blots demonstrated that TNFα-mediated phosphorylation of AKT was also dampened in *F. alocis*-treated cells as compared to media-cultured neutrophils (Figure 6B). The reduced AKT phosphorylation followed the timing of the decreased p38 MAPK phosphorylation, with the phenotype reaching statistical significance only at 6 and 10 h. Like the p38 MAPK phenotype, the lowered AKT activation was dependent on interaction with viable *F. alocis* (Figure 6C) and was not mediated solely through ligation of the TLR2/6 receptor (Figure 6D). Also downstream of p38 MAPK phosphorylation is the activation of translation machinery such as the S6 ribosomal protein (25). Thus, the phosphorylation of S6 was tested in whole cell lysates from neutrophils pre-treated with *F. alocis* prior to TNFα stimulation (Supplemental Figure 1). Densitometry analysis showed that the activation of S6 in response to TNFα was significantly dampened in neutrophils pre-treated with *F. alocis* for 6 h in comparison to media treated controls. While this trend continued in the 10-h pre-treatment condition, it did not reach statistical significance. Together, these results demonstrate that *F. alocis* actively modulates the TNFα signaling pathway by dampening the activation of p38 MAPK and its downstream effectors, AKT and S6 ribosomal protein.

### Functional Effects of *F. alocis'* Inhibition of TNFα-Mediated p38 Phosphorylation

**Figure 7:**
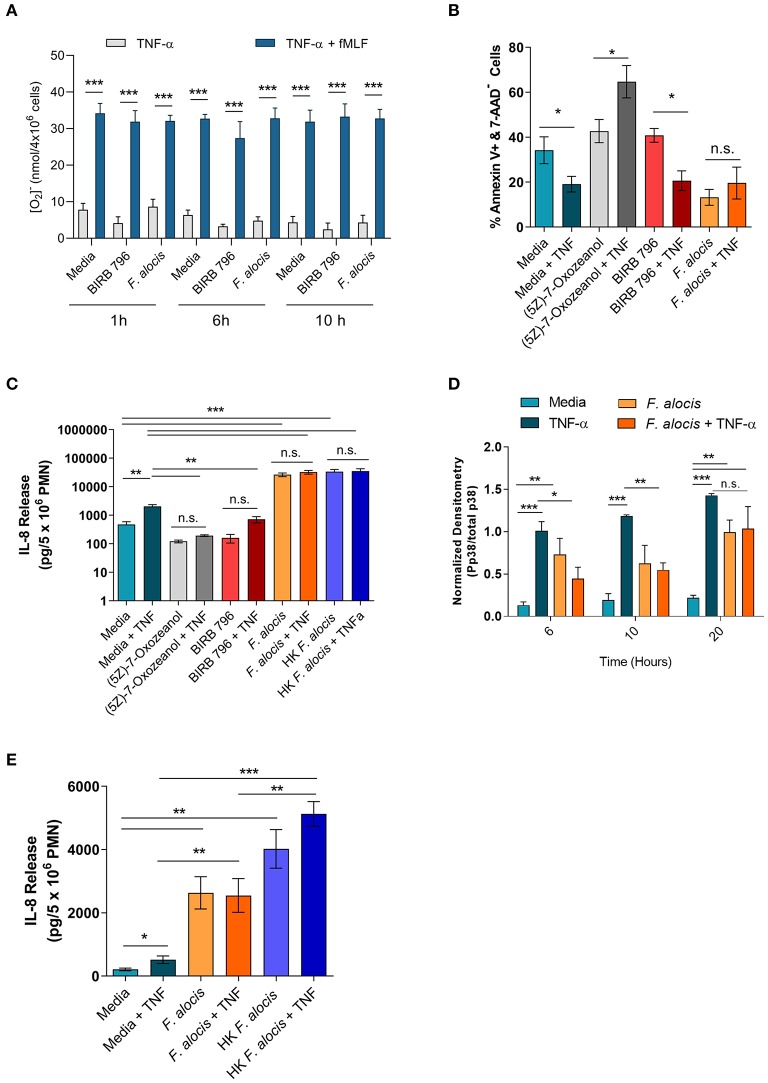
*F. alocis* effect on TNFα-induced functional responses. The ability of TNFα to prime media, BIRB 796 or *F. alocis* pre-treated neutrophils was assessed as the production of superoxide release **(A)**. Cells were cultured in media, pre-treated with *F. alocis* for 1, 6, and 10 h, or the p38 inhibitor BIRB 796 (60 min) followed by TNFα alone or TNFα + fMLF. Results from three independent experiments are shown as the mean ± SEM superoxide production from basal TNFα stimulation or from cells primed with TNFα followed by fMLF stimulation. To test apoptosis, neutrophils were cultured in media or pre-treated with the TAK1 inhibitor (5Z)-7-Oxozeanol (30 min), BIRB-796 (60 min), or *F. alocis* for 6 h followed by a 12-h TNFα stimulation **(B)**. The apoptosis data are plotted as the mean ± SEM percent of early apoptotic cells (Q3: Annexin V+, 7-AAD-) from three independent experiments. IL-8 production was also tested in neutrophils described above as well as neutrophils pre-treated with heat-killed *F. alocis* for 6 h prior to a 12-h stimulation with TNFα **(C)**. The cytokine data are graphed as the mean ± SEM IL-8 release from five independent experiments. The duration of *F. alocis*' inhibitory effect on TNFα-mediated p38 phosphorylation was tested at 6, 10, and 20 h **(D)**. Normalized western blot densitometries are summarized from three independent experiments. Finally, IL-8 production was also tested in neutrophils cultured in media or pre-treated with live or heat-killed *F. alocis* for 6 h prior to a 4-h stimulation with TNFα **(E)**. Data is graphed as the mean ± SEM IL-8 release from four independent experiments. N.s, no significance, *p < 0.05, **p < 0.01, ***p < 0.001.

TNFα stimulation can prime the ROS response of neutrophils, extend their lifespan, and induce cytokine production [reviewed in (56)]. To determine if the interference of TNFα signaling resulted in any phenotypic changes, these three TNFα-mediated functional responses were tested on cells cultured with *F. alocis* for 6 and 10 h prior to TNFα stimulation. The RNA seq screen showed that the mRNA levels of some NADPH oxidase components were affected; thus, before testing the ROS priming response, the protein expression of two of the subunits p47phox (NCF1) and p67phox (NCF2) was determined (Supplemental Figure 2). The RNAseq screen showed the gene expression of p67phox was unchanged when the cells were challenged with *F. alocis*, but the gene expression of p47phox was significantly decreased in *F. alocis*-treated cells (Table 3). At the protein level, there was no significant difference between media cultured neutrophils and those exposed to *F. alocis* at any time point tested for either p47phox or p67 (Supplemental Figure 2). Stimulation with TNFα also had no effect on either subunit's protein expression in media-cultured neutrophils or those exposed to *F. alocis*, demonstrating that any changes observed in the ROS priming response could not be due to differences in the availability of NADPH oxidase components. The basal extracellular superoxide production was similar in cells cultured in media or in media with p38 inhibitor BIRB-796 or *F. alocis* for 1, 6, and 10 h (Figure 7A). However, when BIRB-796 and *F. alocis* pre-treated cells were primed with TNFα followed by stimulation with fMLF, the superoxide production was comparable to that of neutrophils cultured in media prior to the TNFα priming. Thus, we conclude that inhibiting p38 activation through a chemical inhibitor or *F. alocis* does not affect the ability of TNFα to prime neutrophils' ROS response.

In human neutrophils, TNFα stimulation activates MAPK kinase kinase, TAK1 (also known as MAP3K7), which leads to the downstream phosphorylation of ERK1/2 to delay apoptosis and the phosphorylation of p38 to induce cytokine production [(25, 55), Supplemental Figure 5]. First, the effect of *F. alocis*-impaired p38 activation was tested on apoptosis. Neutrophils were cultured in media or media with a TAK1 inhibitor (5Z)-7-Oxozeanol (30 min), a p38 inhibitor BIRB-796 (60 min), or *F. alocis* for 6 h, followed by ± TNFα stimulation for 12 h (Figure 7B, Supplemental Figure 3). Based on Annexin V and 7-AAD staining, TNFα stimulation of cells cultured in media was able to decrease the number of apoptotic cells compared to cells left in media alone. When TAK1 was inhibited by (5Z)-7-Oxozeanol, neutrophils became apoptotic, especially when treated with TNFα. Neutrophils pre-treated with BIRB-796 behaved similarly to media-cultured neutrophils, where TNFα stimulation is pro-survival because inhibition of p38 does not affect the TNFα-ERK1/2 mediated delay in apoptosis. Interestingly, pre-treatment of neutrophils with *F. alocis* alone resulted in a decrease in apoptotic cells, which was not reduced further with TNFα stimulation. Apoptosis was also assessed in cells pre-treated with *F. alocis* for 10 h prior to the 12-h stimulation with TNFα, and results matched the 6-h pre-treatment (Supplemental Figure 4). Together, this data reinforces the finding that only ERK1/2 signaling is important in TNFα-induced neutrophil survival and that *F. alocis* is selectively inhibiting p38 MAPK.

TNFα stimulation can also induce the production of cytokines and chemokines such as interleukin (IL)-8. Thus, the release of IL-8 was tested in the supernatants of cells cultured with media, (5Z)-7-Oxozeanol, BIRB-796, or *F. alocis* for 6 h followed by +/- TNFα stimulation for 12 h (Figure 7C). As expected, TNFα stimulation of media-cultured neutrophils induced significant release of IL-8. Culturing the neutrophils with the TAK1 and p38 inhibitors alone did not induce IL-8 production; however, TAK1 and p38 inhibition reduced the release of IL-8 by TNFα stimulation. Contrastingly, the *F. alocis* pre-treatment alone caused robust release of IL-8, which significantly surpassed the IL-8 release of TNFα-activated, media-cultured cells. Despite the potent IL-8 production by *F. alocis* alone, the further stimulation of *F. alocis* pre-treated cells with TNFα did not cause significant, additional release of IL-8. To rule out the possibility that *F. alocis* treatment alone exhausted the neutrophils' ability to produce IL-8, we also tested neutrophil IL-8 production after pre-treatment with heat-killed *F. alocis*, which show normal p38 activation in response to TNFα stimulation (Figure 5D). Similar to viable bacteria, the heat-killed bacteria-induced significant IL-8 production from neutrophils, and this production was not significantly enhanced with stimulation by TNFα (Figure 7C). This phenotype was also observed when neutrophils were pre-treated for 10 h with viable or heat-killed *F. alocis* prior to TNFα challenge for 12 h (Supplemental Figure 4), suggesting that despite a defect in TNFα-mediated p38 phosphorylation by the viable bacterium, alternative pathways are activated by *F. alocis* that result in maximal IL-8 production from neutrophils in the time points tested. This observation raised the possibility that the inhibition of p38 phosphorylation by *F. alocis* subsides during the 12-h TNFα stimulation period. Therefore, we tested the phosphorylation of p38 in neutrophils pre-treated with media or *F. alocis* for 6, 10 and 20 h prior to TNFα stimulation for 15 min. The inhibition of p38 phosphorylation by *F. alocis* had dissipated by 20 h, suggesting the MAPK dampening by *F. alocis* is a transient effect (Figure 7D). Finally, to determine if IL-8 production is affected during the period of infection where p38 phosphorylation is dampened, we shortened the TNFα stimulation to 4 h (Figure 7E). Despite the shorter stimulation period, TNFα still caused significant release of IL-8 from media cultured cells. Both viable and heat-killed *F. alocis* induced significant IL-8 production on their own, but their responses diverged after TNFα stimulation. While heat-killed *F. alocis* pre-treated cells produced a greater amount of IL-8 upon addition of TNFα, viable *F. alocis*-treated cells were incapable of generating more IL-8. Combined, this data demonstrates that viable *F. alocis* blocks TNFα-mediated p38 activation to reduce transiently the production of pro-inflammatory cytokines, but this interference has no effect on other TNFα induced effector functions like ROS priming or pro-survival response.

## Discussion

As executioners of the innate immune response, neutrophils are recruited to the gingival tissue to provide the host with protection against infection. However, in active periodontal disease sites, the interaction between neutrophils and the dysbiotic microbial community results in dysregulated inflammation, which is detrimental to the host. Composition analysis of the dysbiotic microbial community identified high concentrations of emerging periodontal pathogens such as *F. alocis*. We recently demonstrated that *F. alocis* survives within human neutrophils by inducing minimal ROS production and blocking granule recruitment to the bacteria-containing phagosome (21). Despite *F. alocis* causing significant changes in the mRNA expression of different neutrophil-derived cytokines and chemokines, lower levels of these inflammatory mediators are released when compared to the response elicited by other oral pathogens (22). Therefore, a systems biology-level approach was used to define global changes in human neutrophil transcriptome modulated by *F. alocis*. This unbiased approach provides insights into how this emerging oral pathogen might undermine the innate immune system and contribute to disease progression. Our results show that among the 71 significant biological processes modulated by *F. alocis*, the highest percent were related to inflammation, signal transduction, immune response, and apoptosis. Furthermore, the KEGG pathway analysis revealed that uptake of *F. alocis* significantly downregulated the expression of genes associated with signal transduction pathways, primarily the MAPK cascade and the TNFα signaling pathways. To the best of our knowledge, our results are the first to show that TNF-α-induced p38 MAPK activation is significantly impaired in *F. alocis-*challenged neutrophils.

Research studies from the last 20 years have corrected the misconception that neutrophils were unable to induce changes in gene expression because they are short-lived, differentially terminated cells with a densely condensed nucleus (57–60). Microarray-based approaches show that significant changes in neutrophil gene expression take place after 3–6 h following microbial uptake (59). Likewise, our results show a significant increase in neutrophil DEGs from 624 genes up to 2671 following 1 and 3 h of *F. alocis* challenge, respectively (Figures 1C,D). By 6 h post bacterial challenge, the number of DEGs continued to rise up to 3,489 with a similar number of upregulated (1,739) and downregulated (1,750) genes (Figures 1C,D). Interestingly, the transcriptome studies performed thus far on neutrophils following bacterial interactions reveal common as well as pathogen-specific transcriptional profiles, providing novel information about the potential pathogenic persona of the microorganism studied (60). For example, *Anaplasma phagocytophilum* induces minimal ROS production by neutrophils and a microarray study following 1.5 up to 24 h post-infection shows that the inability to mount the response is not due to modulation of the genes encoding for the different components of the NADPH oxidase (32). We recently showed that *F. alocis* is phagocytized by human neutrophils but induces minimal ROS production (21). In contrast to the transcriptional neutrophil profile elicited by *A. phagocytophilum*, our RNAseq and qPCR results indicate a significant downregulation of the gene that encodes for one of the cytosolic components of the NADPH oxidase, p47phox, and a significant time-dependent increase in the gene expression of galectin 3 (Figures 1E,F and Table 3). P47phox, together with p67phox and p40phox, form the triad cytoplasmic complex, in a 1:1:1 stoichiometric ratio, which is essential for NADPH oxidase activation (61). Although the minimal ROS induced by *F. alocis*-challenged neutrophils was monitored between 1 and 90 min and the significant decrease in p47phox gene expression was observed at 6 h post-infection, these pathogen-induced changes in the transcript levels could leave neutrophils defective in mounting an appropriate respiratory burst response. Furthermore, cytosolic galectin-3 acts as a negative regulator of ROS production in both human and mouse neutrophils by modulating complement receptor 3 signaling pathway during *C. albicans* infections (48). Our results show a time-dependent increase in galectin-3 gene expression which could be one of the strategies used by *F. alocis* to inhibit ROS production. The mechanisms by which *F. alocis* modulates ROS production, both during early and late time points of infection, is an area under investigation in our laboratory.

To mount an efficacious antimicrobial response inside the neutrophil phagosome, the synergy between an optimal activation of the NADPH oxidase and the fusion of the different neutrophil granule subtypes with the bacteria-containing phagosome is essential (61). Microbial pathogens manipulate either one or both of these antimicrobial processes to evade neutrophil killing (62). The expression of CEACAM3-binding opacity (Opa) proteins on *Neisseria gonorrhoeae* renders the organism susceptible to neutrophil killing. The ability of *N. gonorrhoeae* to switch off the expression of Opa proteins, by phase-variation, prevents azurophilic granule fusion to the phagosome, thus promoting bacterial survival (63). During the phagocytic cup formation, effector proteins secreted by *Yersinia pseudotuberculosis* prevent fusion of specific granules to the forming phagosome in human neutrophils (64). Our transcriptome results show that *F. alocis* challenge significantly downregulated neutrophil processes and signaling pathways involved in the regulation of vesicle-mediated transport, neutrophil degranulation, and cellular pathways involved in transport (Figures 2C, 3B, and Supplemental Table 1). This significant downregulation of vesicular trafficking and phagosome maturation transcripts is consistent with our previous results that *F. alocis* inhibits specific and azurophilic granule recruitment to the bacteria-containing phagosome (21). The mechanisms induced by *F. alocis* to modulate neutrophil vesicular trafficking to the phagosome is an area of active investigation in our laboratories.

Neutrophils isolated from periodontitis patients and from healthy controls were transcriptionally active following 3 h challenge with *Fusobacterium nucleatum*, which is found in high numbers in the subgingival plaque from periodontitis patients (33). In this microarray study, *F. nucleatum* induced significant upregulation of genes encoding pro-inflammatory cytokines and chemokines, and it has been shown that this organism induces the release of high levels of these inflammatory mediators from neutrophils (65). In our study we looked at the transcriptional response at 1, 3, and 6 h while the microarray study with *F. nucleatum* was performed only at 3 h. However, our results show that at 1 h post *F. alocis* challenge there is a significant increase in genes involved in pro-inflammatory cytokines such as IL-1β and IL-6, but those same signaling pathways were significantly downregulated by 3 and 6 h (Figures 3A,B). We recently showed that *F. alocis* induces an early increase in both gene expression and protein release of pro-inflammatory mediators such as IL-1β, TNFα, CXCL1, CXCL8, CCL1, CCL2, and CCL3, but the levels released by human neutrophils are significantly lower compared to the response elicited by *P. gingivalis* and *P. stomatis* (22). These results suggested that *F. alocis* might modulate the protein expression and/or release of these inflammatory mediators by neutrophils, and our current results confirm this hypothesis since *F. alocis* pre-treatment limited the release of IL-8 from TNFα stimulation.

An interesting observation from the RNAseq analysis was that *F. alocis* modulates MAPK signaling pathways. Activation of the different MAPK pathways plays a pivotal role in several inflammatory and antimicrobial functional responses both in macrophages and in neutrophils (29). Pathogenic organisms have evolved different strategies to modulate MAPK activation by releasing bacterial compounds into the host innate immune cell that cause kinase inactivation by proteolysis, post-translational modification at active enzymatic sites, as well as by induction of different phosphatases (28, 29). Either inactivation or sustained activation of the MAPK signaling pathways will lead to a dysregulated immune response. We showed that *F. alocis* challenge induces an early activation of p38 and ERK1/2 in human neutrophils that peaks between 15 and 30 min and decreases following 60 min of bacterial challenge (20). We expanded our initial observation, and the data from the present study revealed that *F. alocis* induces a second phase of MAPK activation in human neutrophils beyond 60 min of bacterial challenge. Besides modulation of MAPK signaling, RNAseq analysis showed that *F. alocis* also induced changes in gene expression associated with GPCR and TNF signaling pathways. In the inflamed gingival crevice environment, neutrophils infected with *F. alocis* will also be exposed to bacterial peptides, such as fMLF, as well as to inflammatory cytokines such as TNFα. Our results show a similar degree of fMLF-stimulated p38 and ERK1/2 phosphorylation in human neutrophils in the presence or absence of *F. alocis* infection. However, when TNFα was used as the second stimulus, *F. alocis*-challenged neutrophils showed a significant decrease in p38 MAPK activation. This modulation of TNFα-induced p38 phosphorylation was not seen when neutrophils were exposed to heat-killed *F. alocis* or to the TLR2/6 agonist, FSL-1, prior to the cytokine stimulation. Furthermore, the combination of *F. alocis* and TNFα had no impact on ERK1/2 activation induced by the pro-inflammatory cytokine. While many pathogenic organisms can modulate MAPK signaling in innate immune cells as a mechanism to increase bacterial virulence, this is the first time this observation has been shown for *F. alocis*.

In human neutrophils, TNFα stimulation results in activation of both p38 and ERK1/2, which are involved in the production of inflammatory cytokines and chemokines independently of NF-kB activation (55). However, blocking TNFα-induced activation of p38 but not ERK1/2 impaired both the transcription and translation of inflammatory cytokines by human neutrophils (25). Our results show that TNFα-induced activation of p38 MAPK is impaired in neutrophils infected with *F. alocis*, the extent to which this affects functional mechanisms was tested and is summarized in Supplemental Figure 5. This manipulation of MAPK signaling pathway by *F. alocis* limited the release of TNF-induced chemokine IL-8 by neutrophils. This phenotype has been described for other periodontal pathogens, which employ multiple mechanisms to manipulate IL-8 production and limit the influx of neutrophils (17). In human neutrophils, stimulation by TNFα also has a pro-survival response. It has been shown that activation of MEK and ERK1/2, which are uncoupled in human neutrophils, participate in the prosurvival effect of TNFα (27). A previous study also showed that TNFα activated both p38 and ERK1/2 in human neutrophils, but that only activation of ERK1/2 was necessary for TNFα-mediated inhibition of caspase-3 activity and the pro-survival effect (66). In our study, *F. alocis* challenge did not affect TNFα-induced ERK1/2 activation, and when apoptosis was tested, the pro-survival effect of the cytokine was not impaired. In fact, stimulation with *F. alocis* alone had a pro-survival effect on neutrophils, which was also reflected in the RNAseq analysis where *F. alocis* up-regulated anti-apoptotic signaling pathways in neutrophils.

It has been shown that phagocytosis of pathogenic bacteria such as *Staphylococcus aureus* and *Streptococcus pyogenes* induces changes in neutrophil gene expression involved in the acceleration of apoptosis whereas a different transcriptional profile, linked to delay neutrophil apoptosis, is induced following *A. phagocytophilum* and *Francisella tularensis* infection (32, 34, 60). A microarray study showed that *F. nucleatum* induces upregulation of anti-apoptotic genes in human neutrophils (33). Similarly, our RNAseq analysis identified several upregulated prosurvival and downregulated pro-apoptotic differentially- expressed genes in neutrophils after *F. alocis* challenge. Extending neutrophil life span delays cell turnover and prevents resolution of inflammation contributing to disease progression. The mechanisms utilized by *F. alocis* to delay neutrophil apoptosis is an area under current investigation in our laboratory.

In conclusion, our findings show that *F. alocis* induces significant changes in the human neutrophil transcriptome. In particular, biological processes involved with inflammation, signal transduction, vesicular trafficking, neutrophils activation, and apoptosis were significantly regulated. Furthermore, our results show that *F. alocis* modulated both the TNF and MAPK kinase signaling pathways. This resulted in decreased p38 MAPK activation by a secondary stimulus, i.e., TNFα, but not by fMLF. *F. alocis*, by selectively blocking p38 MAPK, but not ERK1/2, by the secondary stimulus TNF, will potentially maintain a delay of neutrophil apoptosis while dampening the release of inflammatory mediators.

## Data Availability Statement

The datasets for this study can be found in the GEO under accession number GSE137351.

## Ethics Statement

The studies involving human participants were reviewed and approved by Institutional Review Board of the University of Louisville. The patients/participants provided their written informed consent to participate in this study.

## Author Contributions

IM, AV, and MR performed the experiments, participated in data analysis, and interpretation. IM also performed the MetaCore software analysis, data interpretation, and was involved in drafting of the manuscript. ER performed the RNAseq bioinformatics analysis, the PCA, and volcano plots figures. XL participated in RNAseq data analysis and generating a list of differentially expressed genes and figures. SW performed the library preparations and sequencing run of the samples. RL contributed to study design and revision of the manuscript. SU performed the study design, data interpretation, drafting and critical revision of the manuscript, obtained funding, and study supervision.

### Conflict of Interest

The authors declare that the research was conducted in the absence of any commercial or financial relationships that could be construed as a potential conflict of interest.
