# Supplementary material for: Whole Transcriptome Analysis Reveals That Filifactor alocis Modulates TNFα-Stimulated MAPK Activation in Human Neutrophils
Source: Front Immunol. 2020 Apr 16;11:497. doi: 10.3389/fimmu.2020.00497 (PMC7179764; doi:10.3389/fimmu.2020.00497)
Supplement: Supplementary file 1 [file Table_1.pdf]

Supplemental Table 1: Significant biological processes from DAVID analysis categorized by cell function

	**1 Hour**		**3 Hour**		**6 Hour**	
**Inflammatory Response**	**Process**	**P-value**	**Process**	**P-value**	**Process**	**P-value**
	inflammatory response	1.40E-13	inflammatory response	3.30E-15	inflammatory response	4.80E-09
	immune response	7.30E-08	regulation of inflammatory response	1.10E-04	innate immune response	8.50E-06
	positive regulation of inflammatory response	9.30E-04	positive regulation of inflammatory response	5.50E-04	negative regulation of inflammatory response	1.50E-03
	negative regulation of inflammatory response	9.00E-03	negative regulation of inflammatory response	3.60E-03	positive regulation of inflammatory response	1.60E-03
			immune response	6.70E-03	regulation of inflammatory response	1.50E-02
**Response to Microbes**	**Process**	**P-value**	**Process**	**P-value**	**Process**	**P-value**
	cellular response to lipopolysaccharide	1.70E-04	transferrin transport	2.90E-06	response to lipopolysaccharide	1.30E-03
	response to lipopolysaccharide	1.60E-03	response to lipopolysaccharide	3.60E-05	response to muramyl dipeptide	3.80E-03
			lipopolysaccharide-mediated signaling pathway	4.90E-05	defense response	1.10E-02
			cellular response to lipopolysaccharide	3.70E-04	lipopolysaccharide-mediated signaling pathway	1.80E-02
			response to muramyl dipeptide	5.60E-03	cellular response to lipopolysaccharide	1.70E-02

	1 Hour		3 Hour		6 Hour	
**Phagocytosis & Phagosome Maturation**	**Process**	**P-value**	**Process**	**P-value**	**Process**	**P-value**
			cellular response to oxidative stress	1.50E-06	actin cytoskeleton organization	1.10E-02
			phagosome acidification	8.80E-06	phagocytosis	4.00E-03
			intracellular protein transport	3.20E-04	phagosome acidification	6.00E-03
			response to hydrogen peroxide	7.70E-04	response to hydrogen peroxide	7.30E-04
			regulation of macroautophagy	8.60E-04	response to nitrosative stress	4.60E-03
			macroautophagy	2.60E-03	cellular response to oxidative stress	7.20E-03
			autophagy	2.80E-03	positive regulation of autophagy	8.30E-03
			endosome to lysosome transport	5.40E-03	positive regulation of xenophagy	1.10E-02
					negative regulation of inclusion body assembly	1.60E-02
					transferrin transport	7.80E-04
**Chemotaxis**	**Process**	**P-value**	**Process**	**P-value**	**Process**	**P-value**
	neutrophil chemotaxis	5.10E-06	neutrophil chemotaxis	4.70E-05	leukocyte migration	4.80E-04
	monocyte chemotaxis	6.80E-05	chemotaxis	1.30E-04	granulocyte chemotaxis	6.10E-04
	lymphocyte chemotaxis	1.80E-04	positive regulation of neutrophil chemotaxis	6.00E-04	chemotaxis	2.50E-03
	chemotaxis	2.70E-04	leukocyte migration	2.50E-03	neutrophil chemotaxis	3.80E-03
	cell chemotaxis	5.50E-04	positive regulation of cell migration	5.90E-03	positive regulation of neutrophil chemotaxis	2.50E-04
	positive regulation of neutrophil chemotaxis	1.40E-03	monocyte chemotaxis	8.60E-03	positive regulation of cell migration	1.10E-03
	positive regulation of cell migration	3.00E-03				

	1 Hour		3 Hour		6 Hour	
	**Process**	**P-value**	**Process**	**P-value**	**Process**	**P-value**
**Signal Transduction**	positive regulation of ERK1 and ERK2 cascade	8.50E-05	small GTPase mediated signal transduction	2.90E-05	small GTPase mediated signal transduction	3.10E-05
	positive regulation of peptidyl-serine phosphorylation	7.70E-04	intracellular signal transduction	5.70E-04	signal transduction	6.10E-05
	signal transduction	8.40E-04	positive regulation of JNK cascade	5.70E-04	intracellular signal transduction	3.70E-03
	G-protein coupled receptor signaling pathway	2.30E-03	signal transduction	1.10E-03	positive regulation of GTPase activity	4.30E-03
	activation of MAPKKK activity	5.50E-03	TRIF-dependent toll-like receptor signaling pathway	2.90E-03	positive regulation of ERK1 and ERK2 cascade	3.60E-03
	intracellular receptor signaling pathway	7.00E-03	signaling	6.10E-03	inactivation of MAPK activity	1.40E-02
	positive regulation of peptidyl-tyrosine phosphorylation	1.00E-02	regulation of protein phosphorylation	8.90E-03		
			inactivation of MAPK activity	7.00E-03		
			positive regulation of peptidyl-serine phosphorylation	1.00E-02		
			positive regulation of TOR signaling	1.00E-02		

	1 Hour		3 Hour		6 Hour	
**Gene Expression & Transcription Factors**	**Process**	**P-value**	**Process**	**P-value**	**Process**	**P-value**
	negative regulation of transcription from RNA polymerase II promoter	1.70E-05	I-kappaB kinase/NF-kappaB signaling	6.20E-05	negative regulation of gene expression	1.10E-02
	positive regulation of transcription from RNA polymerase II promoter	2.50E-05	negative regulation of I-kappaB kinase/NF-kappaB signaling	7.70E-05	positive regulation of NF-kappaB transcription factor activity	8.40E-04
	positive regulation of tyrosine phosphorylation of Stat3 protein	4.20E-05	positive regulation of NF-kappaB transcription factor activity	1.70E-04	positive regulation of I-kappaB kinase/NF-kappaB signaling	7.10E-03
			positive regulation of I-kappaB kinase/NF-kappaB signaling	1.10E-03	negative regulation of I-kappaB kinase/NF-kappaB signaling	8.30E-03
			positive regulation of protein kinase B signaling	2.30E-03	regulation of I-kappaB kinase/NF-kappaB signaling	1.70E-02
			positive regulation of NF-kappaB import into nucleus	2.70E-03	negative regulation of NF-kappaB transcription factor activity	1.80E-02
			regulation of I-kappaB kinase/NF-kappaB signaling	3.00E-03		
			positive regulation of transcription, DNA-templated	1.00E-02		
			negative regulation of transcription from RNA polymerase II promoter	1.00E-02		

	1 Hour		3 Hour		6 Hour	
	**Process**	**P-value**	**Process**	**P-value**	**Process**	**P-value**
**Cytokines**	chemokine-mediated signaling pathway	4.60E-08	cellular response to tumor necrosis factor	8.30E-06	interferon-gamma-mediated signaling pathway	2.90E-06
	cellular response to tumor necrosis factor	2.00E-07	positive regulation of tumor necrosis factor production	1.60E-05	positive regulation of interleukin-1 beta secretion	3.90E-05
	cellular response to interleukin-1	6.60E-07	chemokine-mediated signaling pathway	3.00E-05	negative regulation of type I interferon production	8.00E-04
	cellular response to interferon-gamma	2.60E-05	positive regulation of interleukin-1 beta secretion	8.30E-05	tumor necrosis factor-mediated signaling pathway	3.40E-03
	tumor necrosis factor-mediated signaling pathway	7.50E-03	cellular response to interleukin-1	4.10E-04	positive regulation of interferon-beta production	6.00E-03
			tumor necrosis factor-mediated signaling pathway	4.10E-03	cellular response to tumor necrosis factor	6.40E-03
			regulation of tumor necrosis factor-mediated signaling pathway	4.30E-03	cellular response to cytokine stimulus	6.50E-03
			negative regulation of transforming growth factor beta receptor signaling pathway	5.10E-03	positive regulation of interleukin-1 beta production	6.50E-03
			positive regulation of chemokine production	5.40E-03	negative regulation of transforming growth factor beta receptor signaling pathway	7.20E-03
			positive regulation of interleukin-8 production	8.60E-03	chemokine-mediated signaling pathway	7.70E-03
					type I interferon signaling pathway	7.20E-03

	1 Hour		3 Hour		6 Hour	
**Protein Folding**	**Process**	**P-value**	**Process**	**P-value**	**Process**	**P-value**
					response to unfolded protein	1.40E-08
					protein folding	1.50E-05
					protein refolding	5.70E-05
					protein ubiquitination	1.60E-02
					protein maturation	1.70E-02
					chaperone-mediated protein complex assembly	8.80E-03
					positive regulation of protein phosphorylation	9.10E-04
**Metabolic Processes**	**Process**	**P-value**	**Process**	**P-value**	**Process**	**P-value**
	positive regulation of nitric oxide biosynthetic process	9.80E-03	glycosphingolipid metabolic process	2.40E-04	glycosphingolipid metabolic process	2.30E-03
	response to amino acid	3.90E-03	cellular response to starvation	1.50E-03	positive regulation of tumor necrosis factor production	3.30E-03
	cellular response to glucocorticoid stimulus	1.10E-03	positive regulation of nitric oxide biosynthetic process	2.80E-03	GTP metabolic process	3.60E-03
	cellular response to corticotropin-releasing hormone stimulus	1.50E-03	cellular response to fatty acid	4.50E-03	proteasomal ubiquitin-independent protein catabolic process	4.60E-03
			UDP-N-acetylglucosamine biosynthetic process	5.60E-03	glycogen biosynthetic process	4.70E-03
			N-acetylneuraminate catabolic process	6.10E-03	carbohydrate metabolic process	1.10E-02
			positive regulation of cholesterol homeostasis	6.10E-03	phospholipid metabolic process	1.10E-02
			ATP metabolic process	6.30E-03	regulation of gluconeogenesis	1.10E-02

			ATP hydrolysis coupled proton transport	6.30E-03	inositol phosphate metabolic process	9.70E-03
			proton transport	2.60E-05	ceramide biosynthetic process	1.40E-02
					N-glycan processing	1.60E-02
					N-acetylneuraminate catabolic process	1.70E-03
					positive regulation of MHC class I biosynthetic process	1.90E-02
	**<u>1 Hour</u>**		**<u>3 Hour</u>**		**<u>6 Hour</u>**	
**Apoptosis**	**Process**	**P-value**	**Process**	**P-value**	**Process**	**P-value**
	negative regulation of apoptotic process	2.00E-05	negative regulation of apoptotic process	2.10E-05	apoptotic process	3.70E-07
	apoptotic process	7.90E-04	apoptotic process	1.60E-04	apoptotic mitochondrial changes	2.60E-03
	activation of cysteine-type endopeptidase activity involved in apoptotic process	1.70E-03	positive regulation of apoptotic process	1.90E-04	extrinsic apoptotic signaling pathway	1.20E-02
			necroptotic process	1.10E-03	activation of cysteine-type endopeptidase activity involved in apoptotic process	4.00E-04
			negative regulation of extrinsic apoptotic signaling pathway via death domain receptors	1.80E-03	activation of cysteine-type endopeptidase activity involved in apoptotic signaling pathway	1.30E-03
			negative regulation of cysteine-type endopeptidase activity involved in apoptotic process	3.20E-03	negative regulation of extrinsic apoptotic signaling pathway via death domain receptors	6.60E-03

			intrinsic apoptotic signaling pathway in response to DNA damage	5.30E-03	regulation of extrinsic apoptotic signaling pathway via death domain receptors	6.70E-03
			regulation of extrinsic apoptotic signaling pathway via death domain receptors	5.40E-03	negative regulation of apoptotic process	1.10E-02
			activation of cysteine-type endopeptidase activity involved in apoptotic process	5.60E-03	positive regulation of apoptotic process	1.10E-03
**Miscellaneous**	**Process**	**P-value**	**Process**	**P-value**	**Process**	**P-value**
	positive regulation of osteoclast differentiation	3.70E-05	positive regulation of osteoclast differentiation	1.60E-03	endoplasmic reticulum calcium ion homeostasis	3.30E-03
	cell adhesion	8.10E-03	positive regulation of calcium ion transport	8.60E-03	cell-cell adhesion	3.50E-03
					secretion by cell	1.60E-02
					proton transport	4.80E-03
					response to iron ion	9.10E-03
					negative regulation of protein serine/threonine kinase activity	9.10E-03
					negative regulation of cysteine-type endopeptidase activity involved in apoptotic process	1.40E-02
					positive regulation of calcium ion transport	4.70E-03

Supplemental Table 2: Genes involved in the Biological Process of Inflammation & Neutrophil activation by MetaCore

		1 Hour		3 Hour		6 Hour	
**Input IDs**	**Network Object Name**	**Log2Fold Change**	**p-value**	**Log2Fold Change**	**p-value**	**Log2Fold Change**	**p-value**
**ENSG00000142208**	AKT(PKB)	-0.268394	0.56125	-1.35053	0.0029	-2.14563	0.00005
**ENSG00000012779**	ALOX5	-0.235755	0.46905	-0.43537	0.17815	-1.30551	0.00025
**ENSG00000115966**	AP-1	0.336876	0.41865	0.492399	0.23355	0.730992	0.0755
**ENSG00000115966**	ATF-2/c-Jun	0.336876	0.41865	0.492399	0.23355	0.730992	0.0755
**ENSG00000143632**	Actin	0.636624	1	-1.3805	1	-0.975943	1
**ENSG00000164742**	Adenylate cyclase	1.20705	1	0.372034	1	0.698685	1
**ENSG00000164742**	Adenylate cyclase type I	1.20705	1	0.372034	1	0.698685	1
**ENSG00000078295**	Adenylate cyclase type II	0.948255	1	0.638634	1	0.867516	1
**ENSG00000138031**	Adenylate cyclase type III	0.214679	0.767	3.72654	0.00005	4.60815	0.00005
**ENSG00000129467**	Adenylate cyclase type IV	-0.906962	0.0755	-0.65237	0.1834	-1.09539	0.02525
**ENSG00000162104**	Adenylate cyclase type IX	0.704655	1	2.14178	0.0006	2.31121	0.0002
**ENSG00000173175**	Adenylate cyclase type V	-4.53876	1	0.0994383	1	-2.46958	1
**ENSG00000174233**	Adenylate cyclase type VI	0.173814	1	0.654431	1	0.28185	1
**ENSG00000121281**	Adenylate cyclase type VII	-0.605382	0.2165	-0.429987	0.3534	-0.103959	0.83925
**ENSG00000155897**	Adenylate cyclase type VIII	-15.5693	1	0.222025	1	2.16771	1
**ENSG00000135046**	Annexin I	0.240346	0.4812	-0.660925	0.0554	-1.59915	0.00015
**ENSG00000120868**	Apaf-1	-0.147656	0.73435	-0.852756	0.06645	-0.543003	0.23705
**ENSG00000138071**	Arp2/3	-0.085798	0.8165	-0.290621	0.39975	-0.611465	0.1097
**ENSG00000015475**	Bid	0.106129	0.8675	1.5263	0.0072	0.85196	0.169
**ENSG00000010671**	Btk	-0.151721	0.70195	-1.02335	0.0073	0.172335	0.69585
**ENSG00000106804**	C5a	-0.670077	0.3237	0.572423	0.49415	0.942053	0.25
**ENSG00000197405**	C5aR	0.291758	0.38	-0.129738	0.7045	-1.24779	0.0008
**ENSG00000108691**	CCL2	2.84034	0.00005	1.91775	0.0006	0.95627	0.06905
**ENSG00000135404**	CD63	0.341514	0.49925	2.20922	0.00005	2.74768	0.00005
**ENSG00000006210**	CX3CL1	0.412438	1	0.807216	1	0.31547	1

		1 hour		3 hour		6 hour	
**Input IDs**	**Network Object Name**	**Log2Fold Change**	**p-value**	**Log2Fold Change**	**p-value**	**Log2Fold Change**	**p-value**
**ENSG00000168329**	CX3CR1	-0.154489	0.76035	-0.579668	0.35505	-0.20214	0.6979
**ENSG00000164305**	Caspase-3	0.0872686	0.80205	0.340595	0.3449	0.796916	0.0377
**ENSG00000064012**	Caspase-8	-0.332073	0.4657	-1.05957	0.0224	-1.78981	0.00025
**ENSG00000172757**	Cofilin	-0.019265	0.96085	-0.29201	0.4632	-1.2949	0.00155
**ENSG00000051523**	Cytochrome b-558	-0.234277	0.64875	0.438144	0.3666	0.201541	0.6868
**ENSG00000172115**	Cytochrome c	0.401099	0.43565	-0.174956	0.7352	-0.420116	0.43985
**ENSG00000168970**	Cytosolic phospholipase A2 beta	-0.649148	0.4939	-0.859994	0.3485	-1.22401	0.20455
**ENSG00000007908**	E-selectin	0	1	15.6737	1	14.7034	1
**ENSG00000163735**	ENA-78	2.78973	1	4.99307	0.0094	4.94474	0.0082
**ENSG00000102882**	ERK1 (MAPK3)	-0.31347	0.50515	-1.46191	0.0036	-1.83436	0.00085
**ENSG00000100030**	ERK1/2	0.111859	0.86515	-1.22384	0.04215	-2.03661	0.00205
**ENSG00000100030**	ERK2 (MAPK1)	0.111859	0.86515	-1.22384	0.04215	-2.03661	0.00205
**ENSG00000168040**	FADD	-1.82699	0.02235	-1.83039	0.0195	-2.24993	0.00495
**ENSG00000171051**	FPR	0.309293	0.4953	0.433394	0.33985	-0.117564	0.80285
**ENSG00000171049**	FPRL1	0.300916	0.7945	0.258945	0.80795	-0.704088	0.52475
**ENSG00000060558**	G-protein alpha-15	0.108801	0.77065	1.07998	0.00265	1.11406	0.0034
**ENSG00000127955**	G-protein alpha-i family	0.571501	1	-12.529	1	-2.51315	1
**ENSG00000114353**	G-protein alpha-i2	-0.334653	0.3361	-0.98326	0.004	-2.03799	0.00005
**ENSG00000088256**	G-protein alpha-q/11	-0.383532	0.6306	-0.040169	0.9544	0.202808	0.78065
**ENSG00000078369**	G-protein beta/gamma	-0.003427	0.99495	0.695778	0.17615	0.279574	0.6152
**ENSG00000177885**	GRB2	0.162711	0.7157	0.776796	0.06805	0.653148	0.16675
**ENSG00000163739**	GRO-1	3.37405	0.00005	1.8271	0.00005	1.77034	0.00005
**ENSG00000081041**	GRO-2	6.33583	0.00005	6.15229	0.00005	6.00946	0.00005
**ENSG00000163734**	GRO-3	4.37038	0.00005	3.77473	0.00005	4.35262	0.00005
**ENSG00000144648**	Galpha(i)-specific peptide GPCRs	-1.48914	0.04105	-1.18726	0.09355	-1.35623	0.064
**ENSG00000174775**	H-Ras	-0.079523	0.88715	-0.100795	0.88615	-2.22536	0.00045

		1 hour		3 hour		6 hour	
**Input IDs**	**Network Object Name**	**Log2Fold Change**	**p-value**	**Log2Fold Change**	**p-value**	**Log2Fold Change**	**p-value**
**ENSG00000100906**	I-kB	0	1	3.16244	0.00005	2.48684	0.00005
**ENSG00000090339**	ICAM1	2.80958	0.00005	2.10994	0.00005	1.37142	0.00605
**ENSG00000108622**	ICAM2	-0.172743	0.70855	0.353612	0.4279	-0.709392	0.13385
**ENSG00000105371**	ICAM4	2.97046	0.0036	2.5412	0.00365	2.49967	0.00435
**ENSG00000111537**	IFN-gamma	-0.411676	1	-1.57507	1	-1.43862	1
**ENSG00000213341**	IKK (cat)	0.0386051	0.9263	-0.201073	0.64655	-0.033242	0.93635
**ENSG00000213341**	IKK-alpha	0.0386051	0.9263	-0.201073	0.64655	-0.033242	0.93635
**ENSG00000104365**	IKK-beta	-0.24989	0.576	1.24499	0.0115	1.03277	0.0572
**ENSG00000136634**	IL-10	-0.882243	0.1873	-0.420915	0.48805	0.508715	0.52685
**ENSG00000109471**	IL-2	0	1	15.6737	1	0	1
**ENSG00000134460**	IL-2 receptor	1.67419	1	4.1224	0.0016	3.33951	0.0022
**ENSG00000100385**	IL-2R beta chain	0.432867	1	0.504894	1	1.06171	0.29405
**ENSG00000113525**	IL-5	0.242808	1	1.40525	1	1.79082	1
**ENSG00000136244**	IL-6	2.99727	0.08685	6.22286	0.0654	5.70078	0.0652
**ENSG00000163464**	IL8RA	-0.746133	0.023	-2.18534	0.00005	-1.99003	0.00005
**ENSG00000180871**	IL8RB	-0.329611	0.40595	-1.94377	0.00005	-2.21343	0.00005
**ENSG00000150995**	IP3 receptor	0.0169103	0.9694	1.96326	0.00005	2.39845	0.00005
**ENSG00000169896**	ITGAM	-0.072381	0.8493	-0.199032	0.59635	-0.635927	0.10645
**ENSG00000160255**	ITGB2	-0.277596	0.3734	-0.768094	0.01255	-2.11987	0.00005
**ENSG00000162434**	JAK1	0.134832	0.66445	0.0062887	0.9844	0.463703	0.14465
**ENSG00000175592**	JunB/Fra-1	0.700587	0.10625	-1.95565	0.00005	-2.27314	0.00005
**ENSG00000170345**	JunD/c-Fos	0.540993	0.1754	-1.63712	0.00005	-0.447981	0.2341
**ENSG00000106683**	LIMK1	-0.488078	0.22955	-1.0831	0.00895	-2.07483	0.00005
**ENSG00000182541**	LIMK2	0.505974	0.16555	0.975505	0.0039	0.210261	0.54895
**ENSG00000111144**	LTA4H	-0.18693	0.6781	-0.627719	0.1415	-0.426102	0.3342
**ENSG00000213903**	LTBR1	-0.811739	0.26125	-1.3433	0.08285	-2.08177	0.0134
**ENSG00000213906**	LTBR2	-1.221	0.41905	-2.23305	0.17685	-3.13535	0.091

		1 hour		3 hour		6 hour	
**Input IDs**	**Network Object Name**	**Log2Fold Change**	**p-value**	**Log2Fold Change**	**p-value**	**Log2Fold Change**	**p-value**
**ENSG00000110514**	MADD	0.0831299	0.8343	-0.649991	0.14525	-0.791325	0.04255
**ENSG00000156575**	MBPH	0.9458	1	1.26023	1	2.31847	1
**ENSG00000169032**	MEK1(MAP2K1)	-0.016729	0.9712	-0.019278	0.9619	-0.485772	0.3074
**ENSG00000169032**	MEK1/2	-0.016729	0.9712	-0.019278	0.9619	-0.485772	0.3074
**ENSG00000126934**	MEK2(MAP2K2)	0.0310253	0.9711	-0.818938	0.27245	-0.478587	0.58065
**ENSG00000095015**	MEKK1(MAP3K1)	0.0913193	0.7935	-0.773331	0.02555	0.30483	0.38005
**ENSG00000109320**	NF-kB	1.4494	0.00125	3.07581	0.00005	2.37882	0.00005
**ENSG00000109320**	NF-kB p50/c-Rel	1.4494	0.00125	3.07581	0.00005	2.37882	0.00005
**ENSG00000109320**	NF-kB p50/p50	1.4494	0.00125	3.07581	0.00005	2.37882	0.00005
**ENSG00000109320**	NF-kB p50/p65	1.4494	0.00125	3.07581	0.00005	2.37882	0.00005
**ENSG00000077150**	NF-kB p52/RelB	1.65511	0.00005	2.46764	0.00005	1.84765	0.00005
**ENSG00000162924**	NF-kB p65/c-Rel	1.09213	0.0241	1.97707	0.00005	1.74788	0.00025
**ENSG00000173039**	NF-kB p65/p65	0.805521	0.075	0.644145	0.14395	-0.831558	0.0612
**ENSG00000109320**	NF-kB1 (p50)	1.4494	0.00125	3.07581	0.00005	2.37882	0.00005
**ENSG00000077150**	NF-kB2 (p100)	1.65511	0.00005	2.46764	0.00005	1.84765	0.00005
**ENSG00000077150**	NF-kB2 (p52)	1.65511	0.00005	2.46764	0.00005	1.84765	0.00005
**ENSG00000100906**	NFKBIA	0	1	3.16244	0.00005	2.48684	0.00005
**ENSG00000146232**	NFKBIE	2.0777	0.0001	2.61055	0.00005	2.19574	0.00005
**ENSG00000006062**	NIK(MAP3K14)	-0.560321	0.4498	-1.84519	0.0133	-1.53898	0.0294
**ENSG00000007952**	NOX1	-1.19698	1	-0.310513	1	-0.99699	1
**ENSG00000051523**	NOX1/p22-phox	-0.234277	0.64875	0.438144	0.3666	0.201541	0.6868
**ENSG00000117592**	NSGPeroxidase	0.215028	0.5748	-0.361838	0.3472	-0.453273	0.27595
**ENSG00000174175**	P-selectin	0.758977	0.2542	-1.2616	0.035	-1.26137	0.05625
**ENSG00000116711**	PA24A	0.253945	0.55755	-1.20128	0.007	-2.08999	0.00125
**ENSG00000184381**	PA2G6	2.83811	0.0049	3.50727	0.00005	3.8014	0.00005
**ENSG00000149269**	PAK1	0.184457	0.58715	0.554972	0.1074	0.320026	0.3308
**ENSG00000180370**	PAK2	0.0139468	0.9761	-0.652408	0.191	-0.826735	0.13585

		1 hour		3 hour		6 hour	
**Input IDs**	**Network Object Name**	**Log2Fold Change**	**p-value**	**Log2Fold Change**	**p-value**	**Log2Fold Change**	**p-value**
**ENSG00000005381**	PERM	0.103196	0.84875	0.0038075	0.99585	-0.866344	0.11665
**ENSG00000138308**	PG12B	-1.56865	1	1.23723	1	1.1968	1
**ENSG00000121879**	PI3K cat class IA	0.205456	0.77335	-0.755579	0.2899	0.305876	0.6754
**ENSG00000121879**	PI3K cat class IA (p110-alpha)	0.205456	0.77335	-0.755579	0.2899	0.305876	0.6754
**ENSG00000051382**	PI3K cat class IA (p110-beta)	0.0572675	0.88035	-0.077703	0.83165	-0.313535	0.38125
**ENSG00000171608**	PI3K cat class IA (p110-delta)	-0.173014	0.60385	-1.013	0.00495	-0.900246	0.012
**ENSG00000145675**	PI3K reg class IA	0.211027	0.69585	-0.252244	0.65605	0.199538	0.70535
**ENSG00000117461; ENSG00000278139**	PI3K reg class IA (p55-gamma)	0.178026	1	0.585365	1	14.7034	1
**ENSG00000145675**	PI3K reg class IA (p85)	0.211027	0.69585	-0.252244	0.65605	0.199538	0.70535
**ENSG00000145675**	PI3K reg class IA (p85-alpha)	0.211027	0.69585	-0.252244	0.65605	0.199538	0.70535
**ENSG00000105647; ENSG00000268173**	PI3K reg class IA (p85-beta)	1.46031	0.26735	1.69027	0.1937	2.09423	0.1278
**ENSG00000101333**	PIB4	-15.5693	1	0.278206	1	1.61796	1
**ENSG00000154229**	PKC-alpha	1.46251	1	1.31425	1	0.964295	1
**ENSG00000166501**	PKC-beta	-0.026308	0.9484	-0.464389	0.27455	-0.237803	0.5807
**ENSG00000163932**	PKC-delta	-0.006682	0.9835	0.907419	0.0052	0.385075	0.2377
**ENSG00000067606**	PKC-zeta	-0.239774	0.5907	-1.19652	0.0118	-1.3945	0.00325
**ENSG00000069764**	PLA2	-0.049767	1	-0.496008	1	-0.257958	1
**ENSG00000069764**	PLA2G10	-0.049767	1	-0.496008	1	-0.257958	1
**ENSG00000123739**	PLA2G12	0.859792	0.40245	-2.39237	1	-1.85832	1
**ENSG00000117215**	PLA2G2D	14.5678	1	15.6737	1	14.7034	1
**ENSG00000100078**	PLA2G3	0	1	15.6737	1	14.7034	1
**ENSG00000105499**	PLA2G4C	1.65877	0.00005	3.85692	0.00005	4.54325	0.00005
**ENSG00000182621**	PLC-beta	0.320995	0.55995	0.0635368	0.91035	-0.749371	0.16965
**ENSG00000182621**	PLC-beta1	0.320995	0.55995	0.0635368	0.91035	-0.749371	0.16965

		1 hour		3 hour		6 hour	
**Input IDs**	**Network Object Name**	**Log2Fold Change**	**p-value**	**Log2Fold Change**	**p-value**	**Log2Fold Change**	**p-value**
**ENSG00000137841**	PLC-beta2	-0.617107	0.1306	-1.58707	0.00005	-2.59408	0.00005
**ENSG00000149782**	PLC-beta3	-0.41557	0.38635	0.297363	0.50225	0.0303082	0.9445
**ENSG00000075651**	PLD1	0.655048	0.2572	2.88132	0.00005	2.86643	0.00005
**ENSG00000129219**	PLD2	-0.420784	0.36975	-1.13418	0.02485	-1.84026	0.0017
**ENSG00000186951**	PPAR-alpha	-0.263617	0.63005	0.456419	0.449	0.30982	0.57365
**ENSG00000124126**	PREX1	-0.044813	0.88785	0.193453	0.5318	0.25828	0.40825
**ENSG00000110876**	PSGL-1	-0.636731	0.0715	-1.4926	0.00005	-1.76971	0.00005
**ENSG00000067900**	ROCK	0.0737406	0.82895	-0.68744	0.03295	-0.369869	0.2781
**ENSG00000067900**	ROCK1	0.0737406	0.82895	-0.68744	0.03295	-0.369869	0.2781
**ENSG00000134318**	ROCK2	0.228265	0.60285	-0.493979	0.3389	-0.3473	0.4248
**ENSG00000136238**	Rac1	-0.058633	0.89405	0.336282	0.4181	0.142861	0.73685
**ENSG00000128340**	Rac2	-0.215299	0.5043	0.527082	0.09755	0.35238	0.2695
**ENSG00000160271**	RalGDS	1.38866	0.0004	2.56647	0.00005	2.03533	0.00005
**ENSG00000067560**	RhoA	0.181352	0.6135	0.393	0.24965	-0.143086	0.7032
**ENSG00000196218**	Ryanodine receptor 1	1.73729	1	0.477101	1	1.04811	1
**ENSG00000092531**	SNAP-23	0.0824211	0.86715	-0.476283	0.29665	0.236652	0.60175
**ENSG00000185338**	SOCS1	-0.443341	0.63635	0.356888	0.62995	2.23929	0.009
**ENSG00000156735**	SODD	-0.259028	0.69995	-1.92762	0.01045	-0.945209	0.15885
**ENSG00000168610**	STAT3	0.318497	0.2861	-0.182545	0.5424	-0.518398	0.0822
**ENSG00000106089**	Syntaxin 1A	-1.49897	0.00725	-3.17114	0.00005	-3.77	0.00005
**ENSG00000103496**	Syntaxin 4	0.915306	0.02265	1.76622	0.00005	1.55602	0.00005
**ENSG00000135823**	Syntaxin 6	-0.697353	0.1095	-1.36091	0.00095	-1.18491	0.0033
**ENSG00000079950**	Syntaxin 7	0.0715859	0.92885	0.412041	0.5591	1.35408	0.05435
**ENSG00000067182**	TNF-R1	-0.474593	0.3514	-0.90111	0.04775	-1.00755	0.06335
**ENSG00000028137**	TNF-R2	-0.1768	0.5946	1.24355	0.00035	0.729701	0.03695
**ENSG00000232810**	TNF-alpha	4.0306	0.00005	2.77055	0.00005	1.15677	0.00165
**ENSG00000226979**	TNF-beta	0.273582	0.5725	1.24517	0.00705	0.212522	0.6612

		1 hour		3 hour		6 hour	
**Input IDs**	**Network Object Name**	**Log2Fold Change**	**p-value**	**Log2Fold Change**	**p-value**	**Log2Fold Change**	**p-value**
**ENSG00000102871**	TRADD	-0.849765	0.05665	-1.15573	0.0059	-0.85951	0.0678
**ENSG00000127191**	TRAF2	-0.070513	0.94135	0.0796205	0.93035	0.548455	0.5636
**ENSG00000131323**	TRAF3	0.0651305	0.87575	2.40231	0.00005	2.67965	0.00005
**ENSG00000263620; ENSG00000220205**	VAMP2	-0.26636	0.79795	-1.81689	0.57355	1.47089	0.0633
**ENSG00000141968**	VAV-1	-0.055979	0.8732	1.09768	0.0008	0.702089	0.03485
**ENSG00000134215**	VAV-3	0.187493	0.6775	0.512374	0.2545	-0.061412	0.896
**ENSG00000100568**	VTI1B	-0.23661	0.7201	-1.13439	0.0526	-0.284376	0.6871
**ENSG00000169896**	alpha-M/beta-2 integrin	-0.072381	0.8493	-0.199032	0.59635	-0.635927	0.10645
**ENSG00000177606**	c-Jun	-0.495397	0.1884	-0.793995	0.03005	-0.218374	0.55605
**ENSG00000175592**	c-Jun/Fra-1	0.700587	0.10625	-1.95565	0.00005	-2.27314	0.00005
**ENSG00000170345**	c-Jun/c-Fos	0.540993	0.1754	-1.63712	0.00005	-0.447981	0.2341
**ENSG00000177606**	c-Jun/c-Jun	-0.495397	0.1884	-0.793995	0.03005	-0.218374	0.55605
**ENSG00000132155**	c-Raf-1	0.127627	0.7162	-0.041701	0.8997	0.248827	0.4648
**ENSG00000116711**	cPLA2	0.253945	0.55755	-1.20128	0.007	-2.08999	0.00125
**ENSG00000165168**	gp91-phox	0.530715	0.25785	1.60592	0.00045	1.62704	0.00055
**ENSG00000007171**	iNOS	-15.5693	1	-12.529	1	2.07421	1
**ENSG00000051523**	p22-phox	-0.234277	0.64875	0.438144	0.3666	0.201541	0.6868
**ENSG00000185386**	p38 MAPK	-2.27641	1	-0.431726	1	-1.38824	1
**ENSG00000112062**	p38alpha (MAPK14)	-0.02206	0.96605	-0.716459	0.1727	-1.45325	0.006
**ENSG00000185386**	p38beta (MAPK11)	-2.27641	1	-0.431726	1	-1.38824	1
**ENSG00000156711**	p38delta (MAPK13)	-0.134623	0.79435	0.596537	0.19465	0.422063	0.37635
**ENSG00000188130**	p38gamma (MAPK12)	0.45834	1	0.110895	1	-0.029106	1
**ENSG00000100365**	p40-phox	0.05836	0.8683	-0.397913	0.2401	0.112339	0.7651
**ENSG00000116701**	p67-phox	-0.011738	0.97455	0.542507	0.11335	0.447425	0.1847
**ENSG00000015475**	tBid	0.106129	0.8675	1.5263	0.0072	0.85196	0.169
